# Efficient Alkyne Semihydrogenation Catalysis Enabled by Synergistic Chemical and Thermal Modifications of a PdIn MOF

**DOI:** 10.1021/acscatal.4c00310

**Published:** 2024-03-14

**Authors:** Jordan Santiago Martinez, Jaime Mazarío, Christian Wittee Lopes, Susana Trasobares, José Juan Calvino Gamez, Giovanni Agostini, Pascual Oña-Burgos

**Affiliations:** † Instituto de Tecnología Química, Universitat Politècnica de València-Consejo Superior de Investigaciones Científicas (UPV-CSIC), Avda. de los Naranjos s/n, Valencia 46022, Spain; ‡ LPCNO (Laboratoire de Physique et Chimie des Nano-Objets), Université de Toulouse, CNRS, INSA, UPS, Toulouse 31077, France; § Department of Chemistry, Federal University of Paraná (UFPR), Curitiba 81531-990, Brazil; ∥ División de Microscopía Electrónica de los Servicios Centralizados de Investigación Científica y Tecnológica de la Universidad de Cádiz (DME-UCA), Facultad de Ciencias, Universidad de Cádiz, Campus Río San Pedro S/N Puerto Real, Cádiz 11510, Spain; ⊥ Departamento de Ciencia de los Materiales e Ingeniería Metalúrgica y Química Inorgánica, Facultad de Ciencias, Universidad de Cádiz, Campus Río San Pedro S/N, Puerto Real, Cádiz 11510, Spain; # ALBA Synchrotron Light Facility, Carrer de la Llum 2-26, Cerdanyola del Valles, Barcelona 08290, Spain

**Keywords:** MOF-mediated, bimetallic nanoparticles, heterogeneous catalysis, alkyne semihydrogenation, nanomaterials characterization

## Abstract

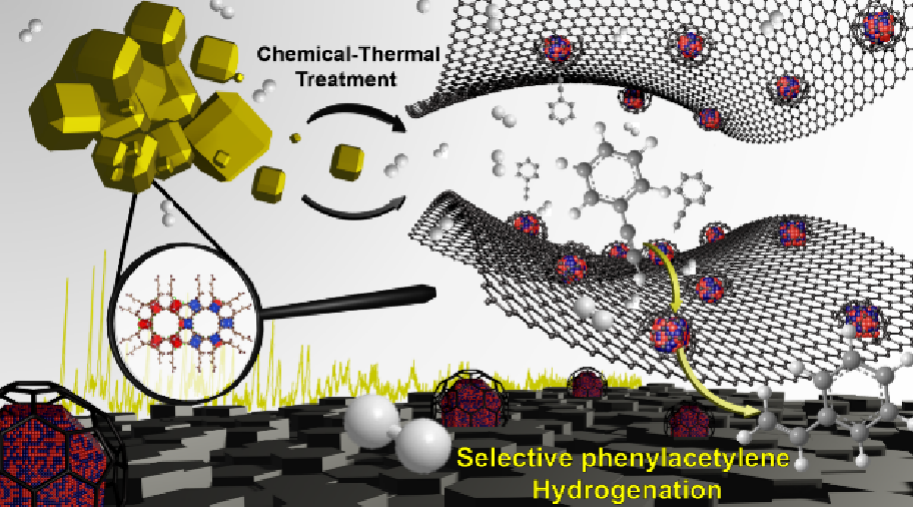

Recently, there has been a growing interest in using MOF templating to synthesize heterogeneous catalysts based on metal nanoparticles on carbonaceous supports. Unlike the common approach of direct pyrolysis of **PdIn-MOFs** at high temperatures, this work proposes a reductive chemical treatment under mild conditions before pyrolysis (resulting in **PdIn-QT**). The resulting material (**PdIn-QT**) underwent comprehensive characterization via state-of-the-art aberration-corrected electron microscopy, N_2_ physisorption, X-ray absorption spectroscopy, Raman, X-ray photoelectron spectroscopy, and X-ray diffraction. These analyses have proven the existence of PdIn bimetallic nanoparticles supported on N-doped carbon. In situ DRIFT spectroscopy reveals the advantageous role of indium (In) in regulating Pd activity in alkyne semihydrogenation. Notably, incorporating a soft nucleation step before pyrolysis enhances surface area, porosity, and nitrogen content compared to direct MOF pyrolysis. The optimized material exhibits outstanding catalytic performance with 96% phenylacetylene conversion and 96% selectivity to phenylethylene in the fifth cycle under mild conditions (5 mmol phenylacetylene, 7 mg cat, 5 mL EtOH, R.T., 1 H_2_ bar).

## Introduction

Among the countless catalytic hydrogenations deep-rooted in the industry, the semihydrogenation of alkynes to alkenes is a critical and challenging process to purify olefin streams, frequently containing alkyne compounds as byproducts. This reaction, by which the trace number of polyunsaturated hydrocarbons are removed to the ppm range, is an essential step in the industrial manufacture of polymer, pharmaceutical, and fragrance intermediates.^1−3^

An efficient semihydrogenation catalyst must balance preventing overhydrogenation of the formed alkene and keeping a satisfactory reaction rate. In that sense, semihydrogenation of alkynes commonly follows the Horiuti–Polanyi mechanism, in which H_2_ is initially adsorbed and dissociated on the catalyst, then an alkyne is adsorbed, and two hydrides are successively added to the unsaturated bond.^4^ Therefore, alkyne and alkene adsorption on the catalyst surface are decisive in product selectivity. Broadly speaking, to successfully direct the selectivity in the alkyne hydrogenation to the alkene, the desorption energy barrier of the π-bond of the alkene should be lower than that of hydrogenation.^5^

Unfortunately, the most significant Pd-based catalysts applied in the semihydrogenation reaction of alkynes have an undesired tendency to overhydrogenation products and polymerization reactions.^6,7^ In fact, due to the strong adsorption of alkenes on large Pd atomic arrangements,^8^ Pd catalysts are highly active but not selective unless they are poisoned, e.g., the Lindlar Pd catalyst (with lead acetate and quinoline). Nevertheless, even industrially relevant Lindlar Pd catalysts suffer from several disadvantages, such as containing environmentally unfriendly lead, low alkene selectivity in the hydrogenation of terminal alkynes, and low stability.^9^

An alternative has been the use of Pd nanocatalysts having elaborate designs. According to density functional theory (DFT) calculations results, the activation barrier of alkene hydrogenation over Pd nanoparticles is relatively low, which leads to alkanes.^5^ Hence, while maximizing activity, Pd must be modified to inhibit the overhydrogenation of alkynes to alkanes. Consequently, several strategies have been proposed to modulate Pd geometry and electron structure. These include combining Pd with other elements (e.g., Ga,^10,11^ In,^12,13^ Zn,^14,15^ Bi,^16^ Cu,^17−19^ Ni,^20^ S,^21^ C,^22−24^ B,^25^ or Ag^26,27^) to prevent the formation of unselective β-palladium hydride and isolate Pd sites to alter the adsorption behavior of intermediates; introducing oxide promoters^28,29^ that deposit preferentially, thus transforming poisoning sites into catalytically active sites; organic-decoration^30−33^ to either fabricate appropriate ensemble structures or tuning chemoselectivity through poisoning; and tailoring metal–support interactions,^24,34−39^ by this means minimizing accumulated species, downsizing Pd nanoparticles, even to single atoms, and favoring alkene desorption.

However, some of the hereabove examples enhance selectivity at the expense of activity and Pd utilization efficiency, while others work in the opposite direction. Therefore, combining several of those approaches in one catalyst is highly desirable, though every so often delicate. Opportunely, the results of several groups signify the importance of metal–organic-frameworks (MOFs) as sacrificial templates for preparing catalysts with unique activities and stabilities, merging several of the previously mentioned features in the same composite. In this line, some recent reviews^40,41^ outline the importance of MOF-mediated synthesis via controlled pyrolysis to produce catalysts that, ideally, preserve exceptionally high porosity and long-range order while displaying reduced particle sizes and encapsulation of the metal nanoparticles in the carbon matrix. In this way, MOF-mediated synthesis has succeeded in accomplishing specific synthetic merits, such as achieving high dispersions for high metal loadings,^42^ unprecedented stoichiometries for mixed metal oxide systems,^43^ and preserving the organic linker as an N-doped carbon.^44^

On the other side, we previously demonstrated how using a soft chemical treatment with aniline and H_2_ can gradually transform a bimetallic MOF constituted by the subunits [Fe^1^_3_(μ_3_-O)(−COO)_6_] and trans-[PdCl_2_(PDC)_2_] (PDC: pyridine-3,5-dicarboxylate) into a bimetallic nanocomposite containing an N-doped graphitic carbon, suitable for both catalytic^45^ and electrocatalytic applications.^46^ While the pyrolytic approaches commonly used result in highly durable materials, the soft chemical transformation we developed enhances the control over the properties of the resulting nanocomposite, thus better preserving the unique features exhibited by MOF precursors, such as high metal dispersion at very high loadings as well as high surface area.

**Scheme 1:**
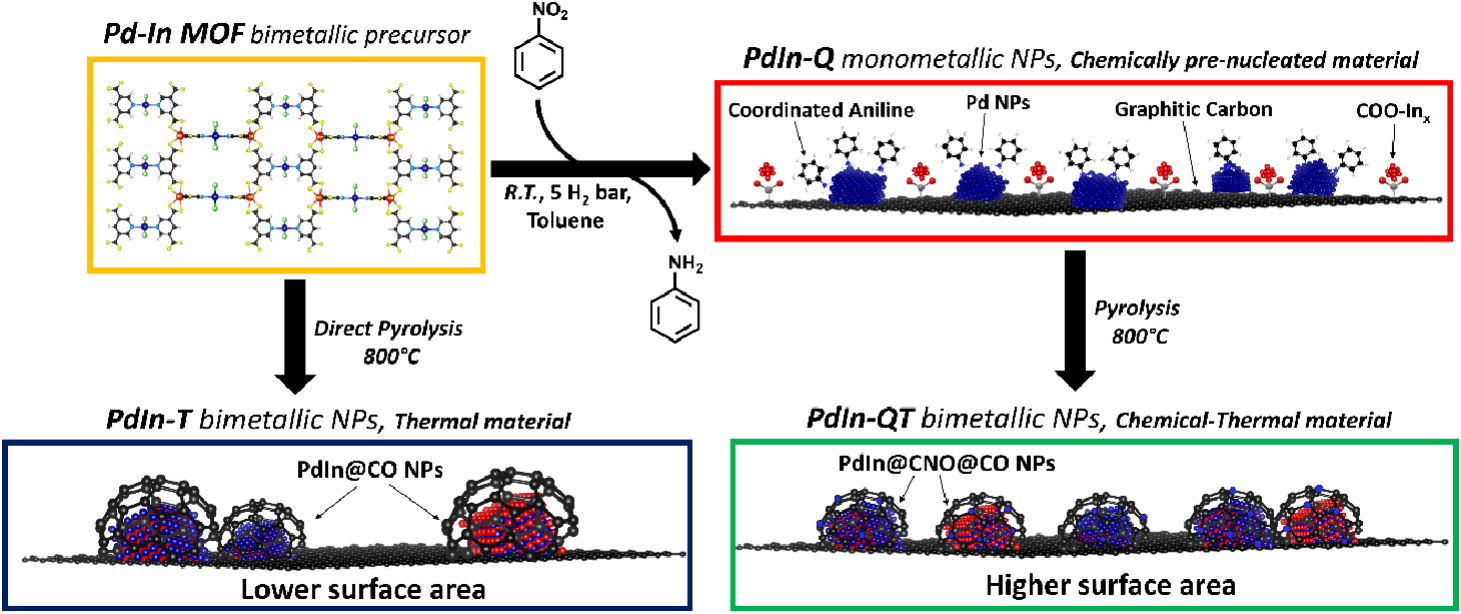
Summary of the Synthetic Strategies Applied in this Work

Subsequently, in this work, combining our previously reported chemical treatment with the well-known pyrolysis on a **PdIn-MOF** (Scheme 1), sharing the above-mentioned structural subunits, allows us to tailor metallic site dispersion, composition, and chemical state. This approach leads to a sophisticated, but very efficient, heterogeneous catalyst composed of PdIn nanoparticles supported on a high surface area N-doped carbon. This nanocomposite presents an enhanced activity and selectivity in alkyne semihydrogenation reaction. Moreover, the present approach could conceivably open a new strategy for generating catalytically active nanocomposites from MOFs.

## Experimental Section

### Catalyst Preparation (see Scheme 1)

#### Preparation of **PdIn-MOF**

**PdIn-MOF** was prepared according to the published procedure.^47^ Typically, a mixture of 0.23 mmol of Pd complex (synthesis detailed in SI; Catalyst Preparation) and 0.53 mmol of InCl_3_ were dissolved under vigorous magnetic stirring in 56 mL of a THF/DMF/H_2_O solvent mixture (28 mL/21 mL/7 mL, respectively). The resulting yellow solution was evenly poured into 10 scintillation vials (5.6 mL in each). Then, the vials were sealed and placed in the oven at 65 °C for 72 h. After cooling the vials at room temperature, the resulting yellow crystals were recovered by vacuum filtration and washed several times with acetone. Finally, the phase purity of the resulting materials was checked by XRD analysis and compared to the reported one.

#### Preparation of **PdIn-T**

200 mg of **PdIn MOF** was transformed by direct pyrolysis in a tubular fixed-bed reactor under N_2_ flow (20 mL·min^–1^) at 800 °C for 2h (ramp 25 °C·min^–1^). Then, the material was cooled down to room temperature under a higher N_2_ flow (40 mL·min^–1^).

#### Preparation of **PdIn-Q**

Inspired by a procedure previously reported by Chaudret,^48^**PdIn-Q** was synthesized from **PdIn-MOF** through controlled reductive decomposition. In this process, an amine (aniline) was employed in the presence of H_2_ (5 bar) at room temperature. However, to make the process even slower, nitrobenzene was slowly hydrogenated in situ into aniline to ensure a gradual addition of the amine to the synthesis mixture. Thus, **PdIn MOF** (400 mg) was placed into a 300 mL hydrogenation reactor with a solution of 80 mmol of nitrobenzene and 80 mL of toluene (yellow mixture). The system was sealed and pressurized at 5 H_2_ bar at room temperature. As demonstrated in our previous research,^45,46^ the proposed approach, which involves the in situ generation of aniline, rather than its straightforward application or use as a solvent, is designed to produce a heterometallic nanocomposite (**PdIn-Q**) that more closely resembles the original MOF and offers improved control of nanoparticle size, resulting in smaller nanoparticles and narrower size distributions. After 24 h of vigorous magnetic stirring, the resulting dark solution was filtrated under vacuum to recover the material. The material was then washed multiple times with methanol and activated at 300 °C under vacuum for 6 h.

#### Preparation of **PdIn-QT**

In order to synthesize **PdIn-QT**, a pyrolytic thermal treatment was applied to the previously depicted material (**PdIn-Q**). Accordingly, 200 mg of **PdIn-Q** (before the activation step under vacuum) was pyrolyzed in a tubular fixed-bed reactor under N_2_ flow (20 mL·min^–1^) at 800 °C for 2 h (ramp 25 °C·min^–1^). Then, the material was cooled down to room temperature with a higher N_2_ flow (40 mL·min^–1^).

### Catalyst Characterization

#### Elemental Analysis (E.A.)

Nitrogen (N), carbon (C), and hydrogen (H) contents of materials were determined with a CNHS EA3000 elemental analyzer from Eurovector (calibrated with Sulphanilamide from Elemental Microanalysis company).

#### Inductively Coupled Plasma Spectrometry (ICP)

iCAP PRO XP inductively coupled plasma atomic emission spectrometer (ICP-AES) was used to determine the metal content of the different materials. Prior to analyses, 20 mg of sample was digested in a mixture of sulfuric acid 98% (4 mL) with few drops of hydrogen peroxide at 100 °C, vigorously stirred for 24 h.

#### Thermogravimetric Analysis (TGA)

This technique has been used to study the decomposition and desorption of molecules from solid materials with temperature. Measurements were conducted in an STA 449F3 Jupiter apparatus from Netzsch. The heating rate was 25 °C·min^–1^ in an air stream, and the temperature ranged from 25 to 800 °C.

#### Powder X-Ray Diffraction (XRD)

X-ray diffraction (XRD) was used to determine the atomic periodical structure of the solids. The X-ray diffraction measurements were acquired according to the powder method, in Bragg–Brentano geometry, using a CUBIX diffractometer from PANalytical operating at 40 kV and 35 mA, and equipped with a PANalytical X’Celerator detector. X-ray radiation with from Cu Kα source was used in the range of 2° to 90° (2θ) with a step of 0.020° (2θ). Experimental diffractograms were compared with those found at the PDF2 database (codes in parentheses).

#### Gas Adsorption Measurements

The relationship between adsorbed gas molecules and the partial pressure at constant temperature was registered in adsorption isotherms. The mathematical adjustment of these isotherms to different theoretical models was used to provide information on the volume adsorbed at a given pressure, enabling the calculation of values, such as the surface area of the solid and the pore volume.

High-resolution CO_2_ adsorption isotherms up to 1 bar were measured in a Micromeritics ASAP 2010 for **PdIn-MOF**. Approximately 150 mg of solid was immersed into a liquid circulation thermostatic bath within a glass sample holder. The adsorption was studied after an overnight activation step at 80 °C under vacuum. Then, the CO_2_ adsorption isotherm at 0 °C was measured. The Dubinin–Astakhov plot for CO_2_ adsorption at 0 °C was used.

On the other hand, nitrogen adsorption isotherms (for BET area) for the MOF-derived composites were recorded in an ASAP 2420 apparatus from Micrometrics at −196 °C from 0 to 1 of relative pressure (*P*/*P*_0_). First, 150 mg of pelletized sample (0.2–0.4 mm) was degassed at 400 °C at ≈5×10^–6^ bar overnight. The BET surface area was calculated by using the Brunauer–Emmet–Teller equation fulfilling the criterion established by Rouquerol et al.,^49^ and micropore volume was calculated by the t-plot method.

#### TEM–STEM Characterization

In transmission electron microscopy (TEM), a focused electron beam is transmitted through a thin specimen, interacting with the sample to provide information on its structure, composition, and crystallography. In our study, TEM, complemented by scanning transmission electron microscopy (STEM), EDS, EELS, and electron diffraction, was crucial for characterizing nanoparticle morphology, size distribution, interplanar distances, and crystalline phases in our samples. In that sense, HR-TEM images were recorded using a Jeol JEM2100F operating at 200 kV. This microscope was equipped with an EDS X-Max 80 detector. Particle size distribution was obtained by fitting nanoparticle size frequency plots with a Gaussian curve using at least 200 size measurements. ImageJ software was used to estimate individual nanoparticle sizes. The analysis of HR-TEM images to identify interplanar distances was done with Gatan Digital Micrograph software. The identification of the crystalline phases was performed comparing the obtained interplanar distances with those provided by the Software X̀Pert HighScore Plus database. Additionally, the EDS X-Max 80 detector (resolution of 127 eV) provided qualitative chemical information about the spatial distribution of each element in the sample. Finally, selected area electron diffraction (SAED) was also used to confirm phase identification (data treatment was again performed with Gatan Digital Micrograph software).

Transmission electron microscopy samples were prepared by placing a drop onto a carbon-coated lacey Cu grid (300 mesh). Scanning transmission electron microscopy (STEM) studies were performed on a double Aberration-Corrected FEI Titan Cubed Themis 60–300, available at the DMEUCA node of the Spanish Unique Infrastructure (ICTS) on Electron Microscopy of Materials ELECMI. The equipment is also equipped with electron energy loss spectroscopy (EELS) and X-ray energy-dispersive spectroscopy (XEDS) spectrometers, GIF Quantum ERS/966, and Super X-G2, thus providing a tool to simultaneously combine spectroscopy and image signals. XEDS and EELS experiments were performed by working in the spectrum imaging (SI) mode,^50^ which allows the correlation of analytical and structural information on the selected regions of the material under study. In this technique, the spectroscopy and HAADF signals were collected simultaneously, while the electron beam was scanned across the selected area of the sample.

STEM–XEDS experiments were recorded using the 4-SDD detectors of the Super X-G2 system of the microscope, using a beam current of 120 pA and a dwell time per pixel of 100 μs. X-EDS maps were obtained analyzing the C–K, O–K, Pd–L, and In–L lines. To improve visualization, the elemental maps were post filtered using a Gaussian blur of 0.8, as provided in the Velox software.

EELS data were acquired using the DUAL EELS acquisition mode, which allows acquiring the core loss spectrum with the accompanying low loss spectrum. A series of EELS spectra (core loss and low loss with an acquisition time of 50 and 0.1 ms, respectively) were acquired using an energy dispersion of 0.25 eV and 50 pA probe current. Chemical information from the samples was obtained by acquiring the C–K, N–K, Pd–M, In–M, and O–K EELS signals. The low loss spectrum was used to realign and calibrate the acquired spectra, and the chemical maps were obtained after quantifying the EELS data using standard methods. In order to identify the different phases present in the sample, the EELS data were denoised using principal component analysis (PCA), and then, the individual spectral responses from the sample were obtained using the independent component Analysis (ICA) method, both available at the Hyperspy open-source program^51,52^ ICA is a mathematical treatment that separates a multivariate signal into additive subcomponents, assuming that these subcomponentes are statistically independent.

#### Field Emission Scanning Electron Microscopy (FESEM)

In FESEM, a focused electron beam scans the sample surface, generating detailed high-resolution characterization images at the nanoscale. FESEM images were acquired using a Zeiss Ultra 55 microscope, operating at 1.0 kV, using powder samples prepared on a sample holder with an S4 double-sided adhesive tape for the dispersion of the sample. This microscope was also equipped with an EDS X-Max 80 detector.

#### Raman Spectroscopy

Raman spectroscopy, a nondestructive technique, investigates molecular vibrations by analyzing inelastic laser light scattering. This allowed us to gain crucial insights into the structural characteristics of the carbonaceous part of the nanocomposites herein reported. A Labram-HR Raman spectrometer (600 mm^–1^ grating, 100 mm entrance slit) coupled to a Peltier-cooled CCD detector and an Olympus BXFM optical microscope was used to acquire Raman spectra. The scattering was produced by excitation at 514 nm by means of a HeNe laser with 0.1 mW excitation power on the samples. The laser beam was focused on the sample at 50 times the microscope objective (numerical aperture = 0.5). Rayleigh scattering was removed by a holographic notch filter, and the Raman spectra were recorded between 200 and 2000 cm^–1^, with a resolution of 0.5 cm^–1^.

#### X-Ray Photoelectron Spectroscopy

In X-ray photoelectron spectroscopy (XPS), X-rays eject electrons from inner atomic shells, raising them beyond the Fermi level (EF). The energy required is called binding energy (B.E.), which is element-specific and influenced by oxidation state and chemical environment. This technique is crucial in catalysis research for its high selectivity in surface layer analysis. In our case, XPS analysis was carried out using a SPECS spectrometer equipped with a Phoibos 150 MCD-9 multichannel analyzer using a nonmonochromatic Mg Kα radiation (50 W, 1253.6 eV). Spectra of powdered samples (∼10–30 mg) were recorded by loading them onto a SPECS stainless-steel sample holder, acquired with constant pass energy values at 30 eV, using a 7 × 20 mm analysis area, at 25 °C, and under an operating pressure of 10^–9^ mbar. Intensities were corrected by the spectrometer transmission function. Spectra peak fitting was performed with CasaXPS software, fixing the main contribution to the C 1s signal at 284.5 eV. A Shirley-type background was subtracted from the signals, and Gaussian–Lorentzian curves were used for binding energy determination of the different contributions for each of the element core levels. Atomic surface concentrations for the different elements were estimated from the integrated intensities of their most intense photoelectron line, corrected by their respective atomic sensitivity factors.

#### X-Ray Absorption Spectroscopy

The energy dependence of X-ray absorption from the inner-shell electrons of the atoms provides valuable insights into the specific elements present in a sample, including their local coordination environment, oxidation state, and electronic structure. Therefore, X-ray absorption spectroscopy (XAS) experiments were conducted at the Pd K-edge (24350 eV) and In K-edge (27940 eV) using the NOTOS beamline at the ALBA Spanish synchrotron facility in Cerdanyola del Vallès, Spain. The initial broad-spectrum beam was narrowed down with a water-cooled Si (111) double crystal, and any unwanted harmonics were filtered out using two mirrors coated with rhodium on silicon. The samples were blended with appropriate quantities of BN and then measured as self-supporting pellets with carefully adjusted thickness to achieve an edge jump of approximately 1. The spectra were acquired in the transmission mode employing ionization chambers as detectors. Pd and In metal foils were employed as references for aligning the data; these were positioned between the I1 and I2 ionization chambers. Multiple spectra were gathered for each sample to ensure the consistency and quality of the signal-to-noise ratio. The reduction of data and the extraction of the function χ(k) were accomplished using the IFEFFIT package.^53^ A corefinement fit was applied to both Pd and In edges for the samples subjected to thermal (**PdIn-T**) and chemical–thermal (**PdIn-QT**) treatments. Additional information regarding the extended X-ray absorption fine structure (EXAFS) fits can be found in the table summarizing the obtained values.

#### Diffuse Reflectance Infrared Fourier Transform Spectroscopy (DRIFTs)

To investigate the catalyst interaction with reactant and product in the alkyne semihydrogenation reaction, we conducted a study by recording IR spectra of the reactant (phenylacetylene) and product (styrene) impregnated on specific samples. The study was carried out in a DRIFT reaction chamber (Harrick, HVC-DRM-5) that was attached to an FTIR spectrometer (Bruker, Vertex70) equipped with an MCT detector. Our aim was to analyze the spectra data to understand the performance of our catalyst with respect to a commercial reference. The FTIR spectra were obtained from 400 to 4000 cm^–1^ wavenumbers with a resolution of 4 cm^–1^ and 32 scans per spectra. In a typical experiment, phenylacetylene or styrene was adsorbed on the catalyst surface of **PdIn-QT** or **Pd/C** commercial catalysts by wet impregnation via ethanolic solution. For that, 50 mg of the reactant was dissolved in 3 mL of ethanol and stirred with 100 mg of catalyst for 30 min under N_2_ atmosphere. Afterwards, the solvent was removed under vacuum in an inert atmosphere, and the material with the preadsorbed probe molecule was placed inside a stainless-steel dome using two ZnSe windows for infrared beam transmission.

### Catalytic Tests

#### General Procedure

Reactions were carried out in a 12 mL glass microreactor equipped with a pressure gauge and a metallic probe for sample collection, on a thermostatic hot plate equipped with a magnetic stirrer (1000 rpm). The alkyne (5 mmol) and the catalyst (substrate/Pd: 323/1 molar ratio, i.e., 7 mg in the case of **PdIn-QT**) were mixed in ethanol (total volume of 5 mL). The reactor was then pressurized at the desired hydrogen pressure (1 bar). Once the reaction was finished, the catalyst was removed by vacuum filtration. The products were identified and analyzed by gas chromatography (Agilent 7890A equipped with an HP5 column: 32 m, 0.25 mm/0.25 μm; and an FID detector). Reactant conversion and product quantification were determined from GC data using calibration curves, dodecane as the internal standard, and the following equations:
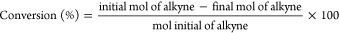

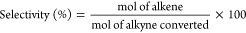

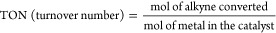

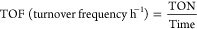

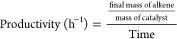


#### Reusability Tests

The catalyst was recovered by vacuum filtration, washed thrice with ethanol (5 mL each), and dried overnight under vacuum. Then, catalytic experiments and analytical protocols followed the above-described methodologies for typical catalytic tests with fresh catalysts.

#### Leaching Tests

Metal leaching was studied by filtering the reaction mixture with 0.45 μm Nylon filters after stopping the reaction at 3 h. This operation has been repeated twice. Thereafter, the filtrate was put back into a clean reactor, and the general procedure for catalytic tests was followed. In addition, this filtrate has also been analyzed by ICP.

## Results and Discussion

### Characterization of the Catalyst Precursor PdIn-MOF

**Figure 1:**
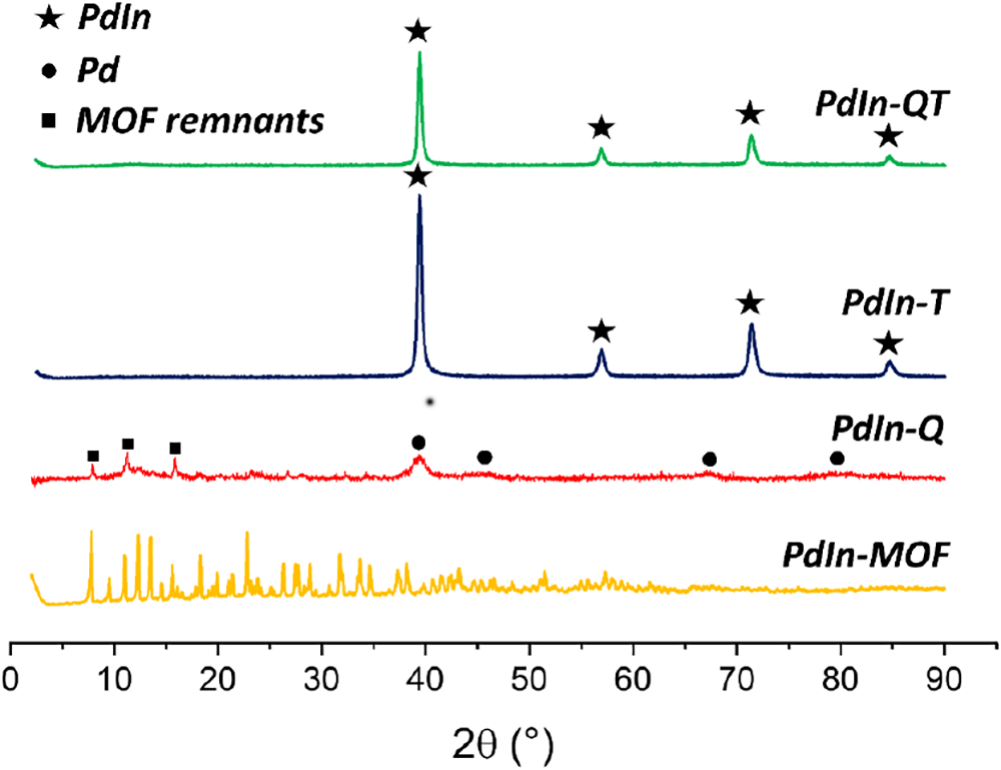
XRD patterns of PdIn synthesized materials. Pd_1_In_1_ (JCPDS: 98-008-3781), Pd (JCPDS: 98-006-0960).

SEM was used to study the morphology of the crystals of the herein synthesized **PdIn-MOF** (Figure S2), finding well-defined, edge-truncated, cubes of 5–40 μm in size, in agreement with the original report.^47^ Additionally, SEM–EDS provided chemical information, confirming the presence of palladium, chlorine, and indium atoms with a Pd/Cl/In molar ratio of 1:2.61:2.33 (Figure S2), fairly close to that reported in the original publication (1:2.67:1.81), and also in agreement with the bulk ICP results (Pd:In = 1:1.90 Table S1).^47^ Furthermore, the XRD analysis depicted in Figure 1 exhibits a diffraction pattern that matches the expected outcome.

Using TGA, the thermal stability of the MOF was assessed. After heating, **PdIn MOF** loses weight in several steps. Below 100 °C, the MOF experiences a weight loss due to the evaporation of the adsorbed solvent molecules. Other losses occur at 200 and 300 °C, probably corresponding to the elimination of coordinated molecules. Above 400 °C, the weight loss can be associated with structural breakdown as the metallo-ligand decomposes, completing the process at 600 °C (Figure S2c). At 800 °C, 39 wt % of residues remained.

Also, the CO_2_ adsorption isotherm of **PdIn-MOF** up to 1 bar was recorded at 0 °C, revealing a CO_2_ uptake of 53 cm^3^·g^–1^ (STP). This value indicates CO_2_ uptakes similar to those reported in the original publication. Finally, Figure S2d shows the corresponding Dubinin–Astakhov plot, which enables the calculation of the specific area of **PdIn-MOF**, i.e., 270 m^2^·g^–1^.

### Characterization of the Catalysts

As previously said, several PdIn nanocomposites were prepared from **PdIn-MOF** by thermal (**PdIn-T**), chemical **(PdIn-Q**), and a combination of chemical and thermal methods (**PdIn-QT**). The chemical composition of these catalytic materials is summarized in Table 1. Note that the use of the thermal treatment involves, in all cases, an increase in the Pd/In molar ratio from 1:2 up to, roughly, 1:1. Moreover, the higher nitrogen content in **PdIn-Q** and **PdIn-QT** already suggests incorporation of anilinic nitrogen into the composite.

**Table 1:** Chemical Composition and N_2_ Adsorption Measurement Results of the Synthesized Materials

material	Pd wt %^a^	In wt %^a^	N wt %^b^	C wt %^b^	BET surface area (m^2^/g)^c^	pore size (Å)^d^
PdIn-Q	14.8	30.9	2.7	24.0	n.d.	n.d.
PdIn-T	27.4	28.0	1.7	13.4	104	53
PdIn-QT	23.6	25.7	2.9	27.9	175	70

a From ICP.

b From EA.

c From N_2_-adsorption isotherm (BET method).

d From N_2_-desorption isotherm (BJH-plot method).

**Figure 2:**
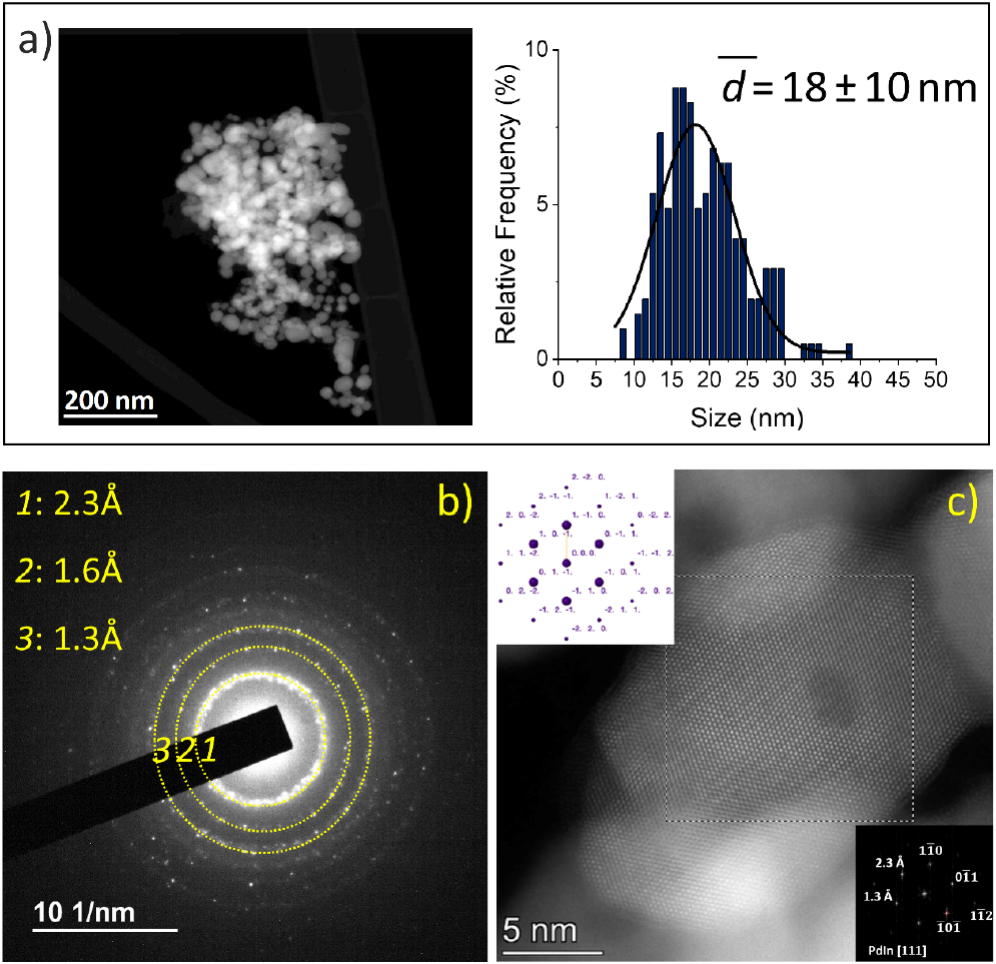
Electron microscopy characterization of **PdIn-T.** (a) Representative HAADF image and nanoparticle size distribution, (b) SAED pattern, (c) representative HR-HAADF STEM image of the **PdIn-T** catalyst. The digital diffraction pattern from the boxed area (shown as inset in the lower right part) could be indexed as that corresponding to the [111] zone axis of Pd_1_In_1_ intermetallic. The image shows in this case contributions of 2.3 Å planes ({110}) at 60° and one at 1.3 Å {112}. Note the perfect match with the simulated diffraction pattern for this phase down the [111] zone axis.

To obtain the **PdIn-T** catalyst, **PdIn-MOF** was activated under a N_2_ flow at 800 °C. This treatment leads to a carbonaceous material where nanoparticles with a broad size distribution are observed by TEM/STEM (18 ± 10 nm, Figure 2a). To analyze the structural data of these catalysts, it is important to consider the inherent complexity of the Pd–In system, which could comprise, apart from the pure metal components, up to 7 other intermetallic phases depicting Pd/In molar ratios ranging from 3 to 0.43. In this situation, the interpretation of the structural data requires the combined use of ICP, XRD as well as TEM/STEM imaging and spectroscopic compositional data.

The XRD diagrams of **PdIn-T** exhibit reflections at interplanar distances that are typical of the {110}, {200}, {211}, and {220} planes of a Pd_1_In_1_ intermetallic (CsCl structure) phase (JCPDS 98-008-3781), in good agreement with the ICP results. Peaks corresponding to other Pd/In compositions are not observed.

Regarding electron microscopy results, reflection rings at 2.3 Å, 1.6 Å 1.3 Å, 1.2 and 1.0 Å could be detected in the selected area electron diffraction patterns of this sample (Figure 2c), which match very closely those expected for the {110}, {200}, {211}, {220}, and {310} planes in a Pd_1_In_1_ alloy.

**Figure 3:**
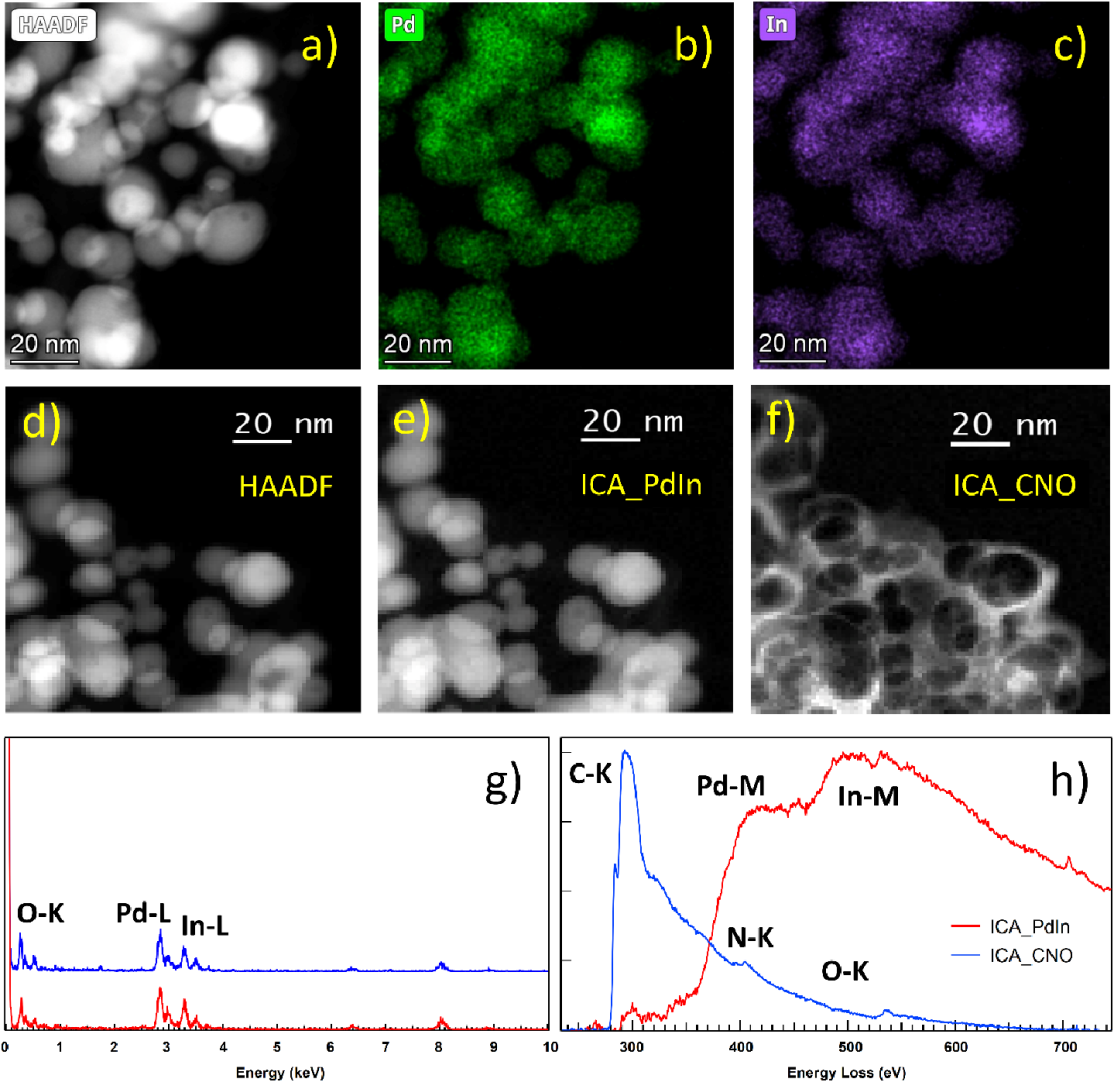
STEM–EDX and STEM–EELS of the PdIn-T sample. Study including a HAADF image (a); the corresponding chemical maps extracted from the STEM–SI–EDS: Pd (b) and In (c); and a representative EDS spectrum (g). STEM–EELS study including a HAADF image (d); and the images corresponding to the different components of the ICA analysis obtained from the whole set of STEM–EELS–SI data (e) and (f). (h) EELS spectra corresponding to the two independent components: a Pd–In component and a C–N–O component.

STEM–EDS and STEM–EELS analyses (Figure 3) confirmed the simultaneous presence of Pd and In in the NPs. Moreover, HR-STEM HAADF images were recorded, which could be unambiguously assigned to the <111> zone axis of the InPd intermetallic (Figure 2c).

From a thermodynamic point of view, Pd_5_In_3_ is the most stable intermetallic. However, considering the free energy of formation of the different Pd–In stoichiometries (Table S2) and the actual availability of reactants characteristic of the T sample (Pd/In molar ratio = 1), Pd_1_In_1_ is the composition allowing the largest free energy evolution during the mixing process (Figure S5). It appears that the changes in free energy that occur during the thermal process are crucial in determining the observed PdIn stoichiometry. However, other factors, such as mechanistic and kinetic aspects, may also play a role in this outcome.

The nature of the carbon remaining in the catalyst after the thermal treatment was first investigated by Raman spectroscopy (Figure S3). The material shows a typical signal from the G band of graphitic carbon at 1599 cm^–1^ and the relative intensity between D and G bands (*I*_D_/*I*_G_ = 0.74) reveals the formation of graphitic carbon with a high number of defects.^54−56^ Interestingly, STEM–EELS SI analyses indicate the presence of two independent components (Figure 3h), one corresponding to the bimetallic nanoparticles (Figure 3e) and a second one due to a thin C–N–O layer (Figure 3f), which surrounds the bimetallic nanoparticles The presence of this outer layer could also be observed in the HR-STEM images of the NPs, as illustrated in Figure S4. Further analysis of this layer revealed a lattice spacing in the order of 3 Å, which is close to, for example, that of (002) planes in C_2_N_2_O.

As for the chemical method, it is inspired by an earlier approach established by Chaudret for the transformation of organometallic complexes into well-defined NPs.^48^ This method involves their controlled decomposition in the presence of a stabilizing ligand (aniline, in our case) under H_2_ (5 bar) at mild or soft reaction conditions (room temperature). As in the first reported example we published, this modified methodology generates in situ the aniline from the hydrogenation of nitrobenzene, providing the reaction mixture with a slow supply of the amine, which also takes part in the deconstruction of the MOF.^45^ In this way, **PdIn-MOF** transforms into a nanocomposite (**PdIn-Q**), including metallic nanoparticles and a graphitic carbonaceous support. However, because of a more gradual deconstruction process of the parent MOF, the NPs are considerably smaller in this case than those obtained via the pyrolytic approach. In Figure S6 two populations can be distinguished by HR-STEM imaging: 1.6 ± 0.7 and 6.8 ± 2.5 nm. STEM–EELS SI and STEM–EDS maps in Figure S7 reveal that Pd is mostly present in the form of pure Pd nanometer-sized nanoparticles. However, a minor contribution of even more highly dispersed pure Pd species is also present. Moreover, STEM–EELS analysis indicates the presence of three independent components: a Pd component, a C–N–O component, and a C–N–In–O component Figure S7e–i). This suggests that In remains in a highly dispersed state, mixed at the atomic level within a support that contains C, N, and O.

In the inset of Figure S7i, the fine structure of the C-edge region in the C–N–In–O EELS component shows an aniline-type carbon. This finding is consistent with the higher concentration of N observed in the sample by EA compared to the **PdIn-T** sample. Additionally, a second pure C component is also present in the support, in the form of a nanometer-thick outer layer. Note that this layer adopts a ribbon-like morphology surrounding the C–N–In–O areas (Figure S7f,h).

These results suggest the presence of carbonaceous MOF remnants containing In, together with Pd nanoparticles, after the chemical treatment, in good agreement with XRD diagrams (Figure 1), which depict the reflections at 2.24, 1.94, 1.37, and 1.17 Å characteristic of (111), (200), (220), and (311) Pd^0^. Ultimately, the graphitic nature of the carbon generated during the chemical process has been demonstrated by Raman spectroscopy in Figure S3. Spectra from **PdIn-Q** show the typical G band of graphitic carbon at 1599 cm^–1^. The relative intensity between D and G bands (*I*_D_/*I*_G_ = 0.37) reveals a low defect concentration in this case.^54−56^

It is clear from these results that the mild conditions prevailing during the chemical (Q) treatment allow for the decomposition of the starting MOF, but do not provide the necessary mobility of the metallic species required to promote their mixing into an intermetallic phase. Mostly, Pd bonding to the MOF structure seems affected by the decomposition process. Detachment and sintering of a fraction of Pd leads to small, pure Pd nanoparticles. On the contrary, most In still remains attached to C, N, and O, highly dispersed all over a graphitic carbon mantle resulting from the partial decomposition of the MOF. The higher temperature used during the T treatment provides the proper conditions to proceed with a more intense MOF decomposition and drive the mixing of the two metals.

**Figure 4:**
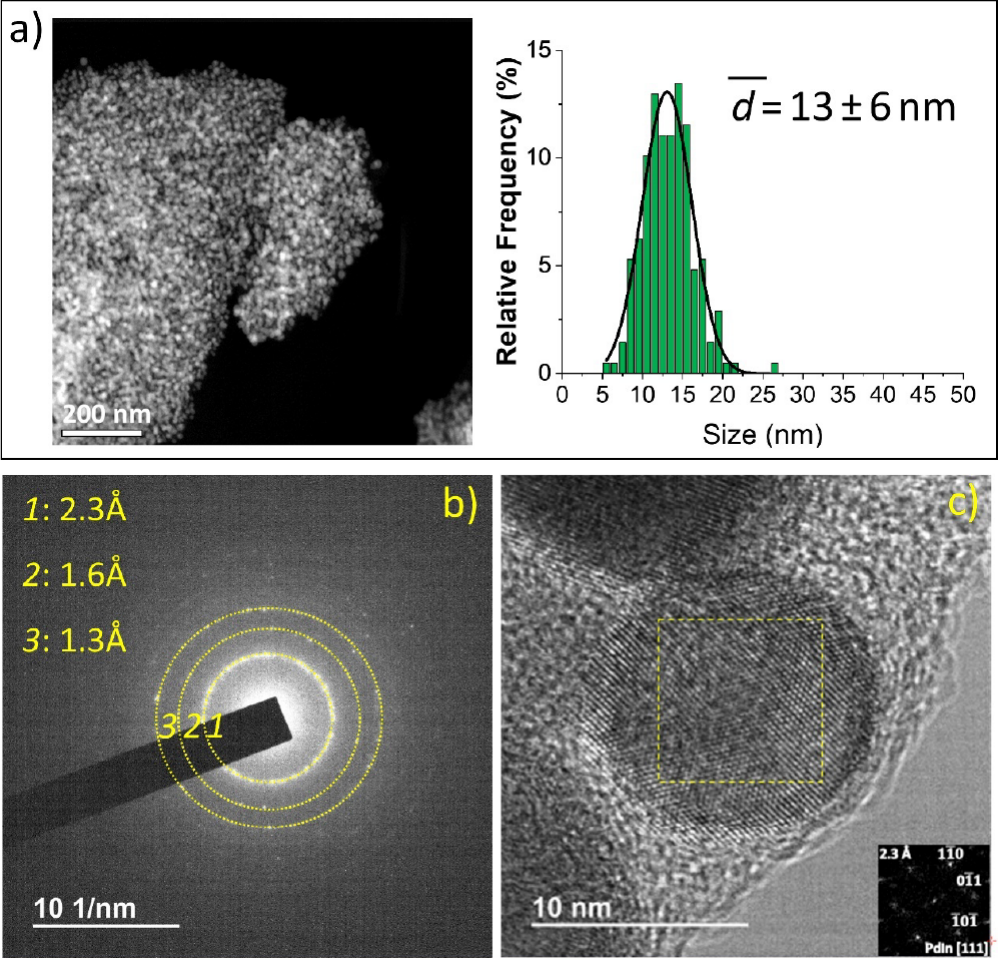
Electron microscopy characterization of the **PdIn-QT** sample. (a) Representative HAADF image and nanoparticle size distribution, (b) SAED pattern, and (c) representative HR-TEM image of the **PdIn-QT** catalyst. The digital diffraction pattern from the boxed area (shown as inset in the lower right part) could be indexed as that corresponding to the [111] zone axis of Pd_1_In_1_ intermetallic.

**Figure 5:**
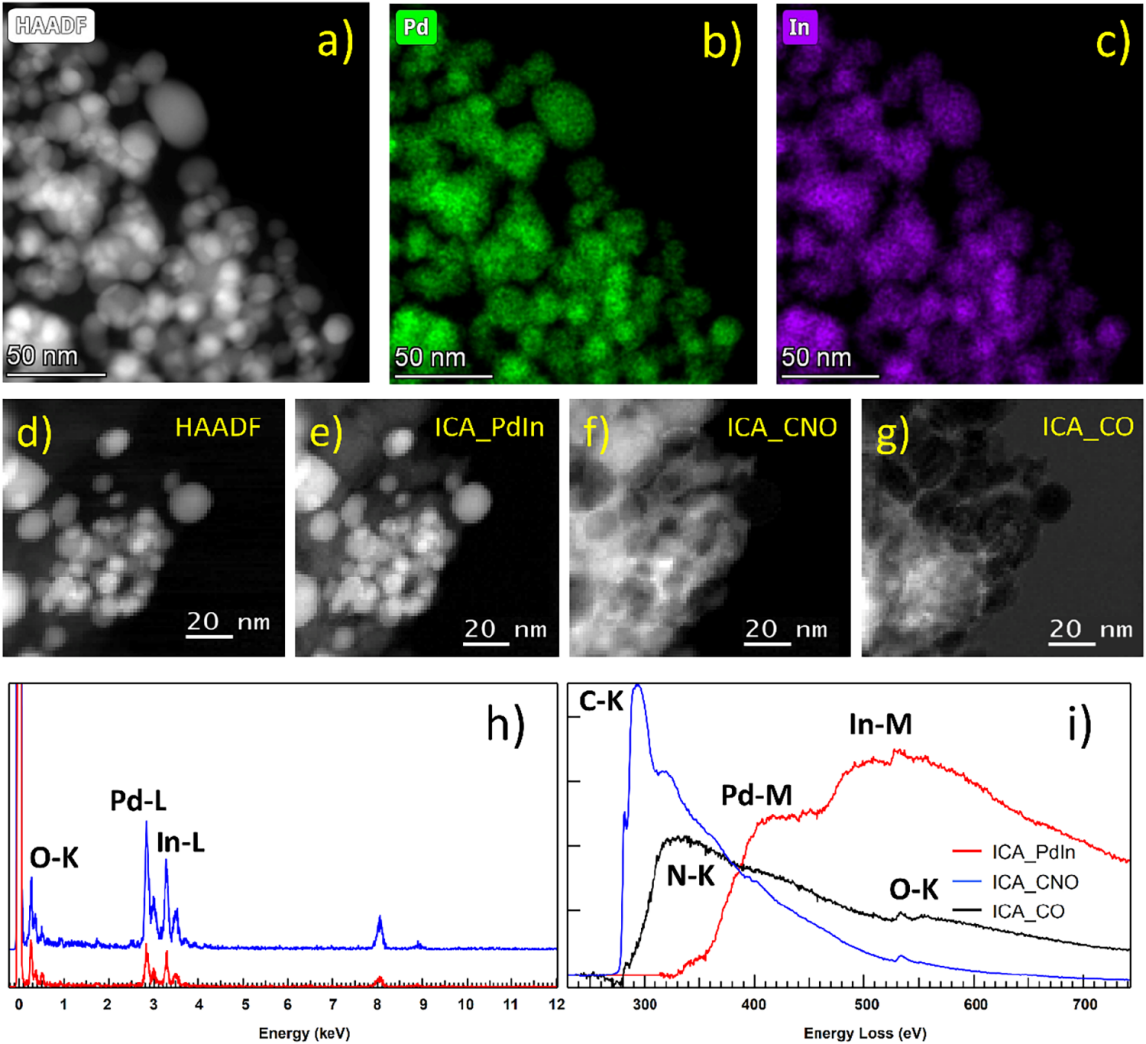
STEM–EDX and STEM–EELS of PdIn-QT sample. (a) HAADF image and the corresponding chemical maps extracted from the STEM–SI–EDS, (b) Pd and (c) In, and (h) a representative EDS spectrum. STEM–EELS study including (d) a HAADF image; and the images corresponding to the three components of the ICA analysis of the whole set of STEM–EELS–SI data (e), (f), and (g). (i) EELS spectra corresponding to the three independent components, a Pd–In component, a C–N–O component, and an external C–O component.

Finally, the combination of both, first the chemical process and subsequently the pyrolytic treatment (**PdIn-QT**), enabled the generation of a third carbonaceous composite, whose metal nanoparticles exhibit a size distribution of 13 ± 6 nm (Figure 4), which lies somewhere in between those attained by the individual treatments previously described. The bimetallic nature of the nanoparticles is well supported both by XRD and electron diffraction patterns, which evidence once more the features characteristic of a Pd_1_In_1_ (CsCl structure) intermetallic phase (Figures 1 and 4). The FFT analysis of the HR-TEM image in Figure 4c confirms this interpretation. Remarkably, STEM–EELS SI and STEM–EDS studies of this sample, Figure 5, reveal that, with this novel methodology to transform the MOF, PdIn nanoparticles arise. These NPs are surrounded by a double C-containing layer (Figure 5f,g). A first one, mixing C–N–O, as in the **PdIn-Q** case, and a second, outer one, containing only C and O. Analysis of the HR-TEM images of this sample (Figure S8) indicates the presence of lattice spacings in the order of 3 Å in the inner C-containing layer and of 4.3 Å in the outer one. These could be ascribed to a C_2_N_2_O-like and a graphitic-like phase with a slightly expanded (001) interlayer spacing, respectively.

Raman spectra of this material shows the typical graphitic carbon G band at 1599 cm^–1^, as in the other materials. Additionally, the ratio *I*_D_/*I*_G_ = 0.75 indicates the order of the carbon is very low, as in the **PdIn-T** material.

**Figure 6:**
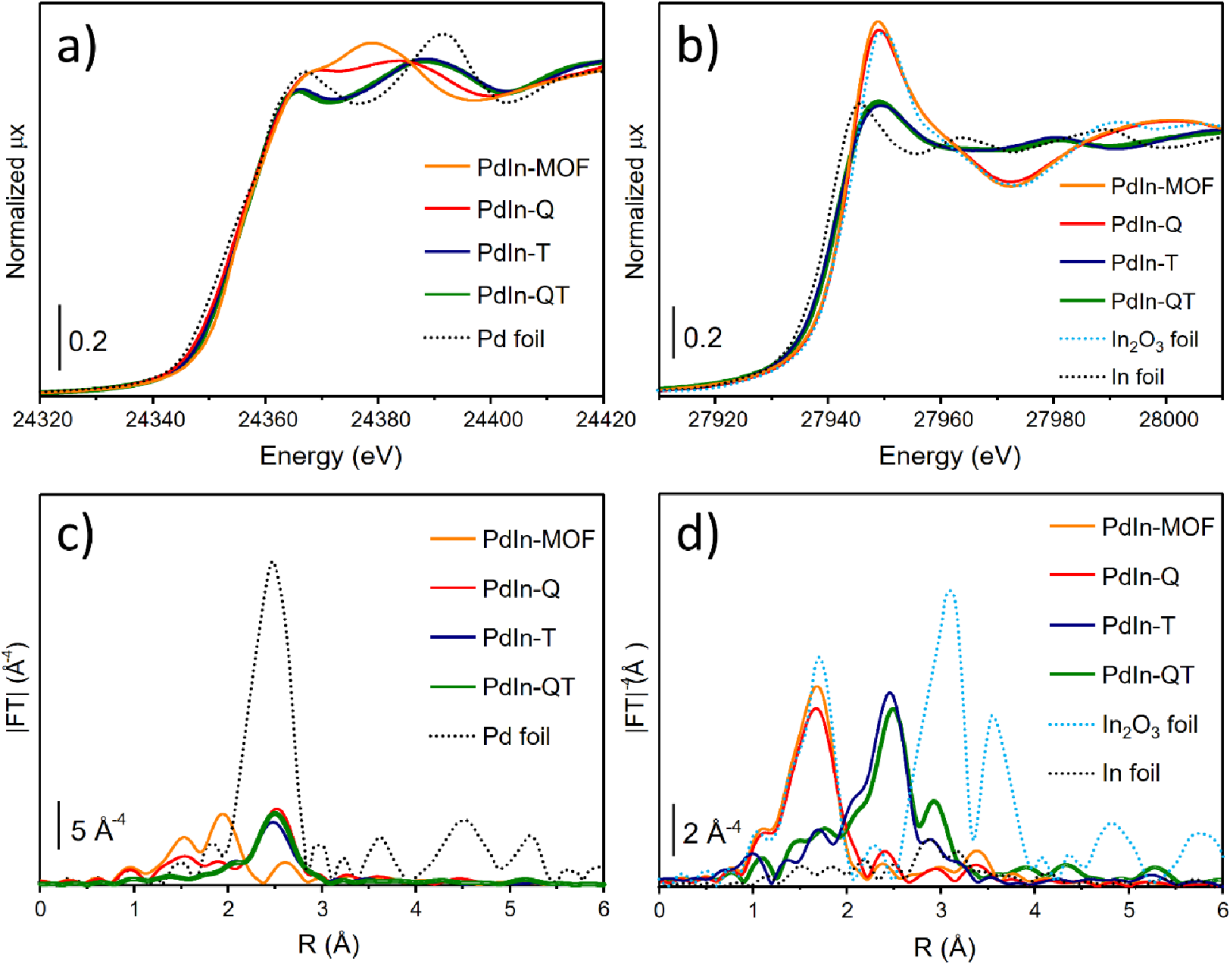
XANES spectra at the Pd (a) and In (b) K-edges, and k^3^-weighted |FT| EXAFS spectra of Pd (c) and In (d) data of MOF-derived PdIn samples.

Aiming at reinforcing the conclusions established by electron microscopy, XRD, and Raman, X-ray absorption spectroscopy was also performed in order to obtain information about the electronic properties and local environment of Pd and In atoms in the bulk of PdIn materials (Figure 6). From Pd and In K-edges XANES spectra, it is possible to observe the evolution of Pd and In atoms from **PdIn-MOF** up to the MOF-derived samples.

Starting with the Pd K-edge, the spectrum of **PdIn-MOF** shows the same position (24354 eV) as PdO and PdCl_2_, indicating Pd atoms are present in the material as Pd^2+^ (Figure S10a). Despite the similarity in edge position, the spectral shape does not resemble those of PdO and PdCl_2_, pointing out that the local environment is different in the sample with respect to the standards. By checking the EXAFS part of the spectrum (orange curve in Figure S10b), two first-shell contributions at 1.55 and 1.89 Å (non-phase-corrected) can be observed, which can be ascribed to Pd–L (L = N/O) and Pd–Cl, respectively, and matches with the Pd neighborhood for the bimetallic precursor shown in Scheme 1. When **PdIn-MOF** is chemically treated (**PdIn-Q,**Figure 6a), its spectrum is readily modified, with the Pd edge position shifting to lower energy and staying between those spectra of Pd^2+^ and Pd^0^ references (24352 eV), indicating a partial reduction of Pd atoms. In fact, a linear combination analysis of the XANES region gives a fraction of metallic Pd of 33%, which confirms the partial reduction of palladium (Figure S11). By looking at the EXAFS spectrum of the **PdIn-Q** sample (Figure 6c), it is possible to observe three main contributions; the first two were tentatively ascribed to Pd–O and Pd–Cl, respectively, while the third one is characteristic of Pd–Pd. The first two contributions lie at the same 1.55 and 1.89 Å positions at the |FT| as those in **PdIn-MOF**, which would imply that some MOF motifs remain in the sample after chemical treatment. This interpretation indeed aligns with the permanence of the MOF observed by XRD (Figure 1). Furthermore, the features observed in both XANES and EXAFS of **PdIn-Q** point out Pd^0^ is present as an *fcc* structure,^57^ which is also in good agreement with our XRD results. Likewise, these results agree with the results of the nanoanalytical STEM–EELS and STEM–EDS study.

On the contrary, the thermal (**PdIn-T**) and chemical–thermal (**PdIn-QT**) derived materials show spectra with a Pd edge position at ∼23351 eV, and a different spectral shape with respect to the Pd references. This energy position and spectral shape are similar to that of PdIn alloy with a CsCl structure reported in the literature,^58,59^ in accordance with XRD, TEM, and EDS results. Additionally, |FT| of reduced samples (Figure 6c) show a main Pd–Pd contribution between 2.0 and 3.0 Å (non-phase-corrected), with significantly lower intensity with respect to metallic Pd, which is related to the highly dispersed character of the nanoparticles formed. It is also possible to observe that Pd–Pd peaks of **PdIn-T** and **PdIn-QT** are shifted to lower *R* values with respect to the **PdIn-Q** and Pd^0^ spectra, which can be attributed to their different crystalline structure (*Pm*-3m vs *Fm-*3̅m).

Regarding the In K-edge (Figure 6b), the spectrum of the initial sample shows the edge position at the same energy as well as spectral features as the In_2_O_3_ reference, which is expected since the coordination environment of In atoms in the MOF resembles that of indium oxide clusters. The XANES of the material after chemical treatment (**PdIn-Q**) is not affected as in the case of Pd K-edge, which suggests the Pd environment is much more affected than In. From these results, it is unclear whether pure In_2_O_3_ clusters are being formed or whether the In is still bonded to carboxylate species as in the MOF structure. However, the presence of a C–N–In–O component in the EELS SI study suggests that the latter might be true.

When the samples are submitted to thermal and chemical–thermal treatment, the whiteline intensity of those spectra decreases and the absorption edge shifts to lower energies (27941 eV), indicating the reduction of In atoms. However, the shape of the spectra does not resemble that of In metal but that of PdIn in a CsCl-type structure.^57^ The moduli of Fourier transform at the In K-edge spectra (Figure 6d) shows the clear disappearance of the In–O contribution present in the **PdIn-MOF** and **PdIn-Q** samples and the appearance of In–Pd and In–In metallic contributions in the **PdIn-T** and **PdIn-QT** samples.

EXAFS fittings are summarized in Tables S3 and 2 (see Figures S12 and S13 for plotted curve fittings). As commented above, **PdIn-MOF** (Table S3) shows two contributions at the |FT| of the Pd K-edge spectrum, with *N*_Pd–L_ = 1.8 (*R*_Pd–L_ = 1.99 Å) and *N*_Pd–Cl_ = 1.8 (*R*_Pd–Cl_ = 2.307 Å), which are in good agreement with the theoretical coordination numbers of 2 for Pd–L and Pd–Cl bonds in MOF structure. |FT| at the In K-edge spectrum shows a first shell related to In within the MOF cluster with *N*_In–O_ = 7 (*R*_In–O_ = 2.159 Å). After chemical treatment (**PdIn-Q**), the Pd–L and Pd–Cl coordination numbers and bond distances remain practically the same, with a slight decrease in N_Pd–Cl_, and the maintenance of *N*_In–O_. However, *N*_Pd–Pd_ = 4.2 (*R*_Pd–Pd_ = 2.772 Å) is now present, confirming the formation of small *fcc* Pd nanoparticles. The thermal (**PdIn-T**) and chemical–thermal (**PdIn-QT**) samples present highly similar Pd environments, with *N*_Pd–In_ = 5.7 (*R*_Pd–In_ = 2.728 Å) and 5.5 (*R*_Pd–In_ = 2.745 Å), respectively. Despite presenting a value of ∼5 (similar to the 4.2 of the **PdIn-Q** sample), it should be stressed that the CsCl structure displays a first shell of 8 vs 12 from *fcc*. Apart from the main Pd–In contribution, these samples present small In–L and In–In contributions, which we tentatively ascribe to the interaction of In with the carbon patches of the support and a small clustering or segregation of In atoms within the nanoparticle (second shell), respectively.

**Table 2:** Summary of EXAFS Fits of **PdIn-T** and **PdIn-QT** Samples Performed on Pd and In Edges at the Same Time^a^

sample	path	*N*	Debye–Waller σ^2^ (Å^2^)	*R* (Å)	Δ*E* (eV)	R-factor
**PdIn-T**	In–O	1.4(5)	0.009(7)	2.12(2)	0.7(7)	0.017
	Pd–In	5.7(3)	0.0101(4)	2.728(4)	0.9(4)	
	In–In	1.4(8)		3.28(4)	0.7(7)	
**PdIn-QT**	In–O	1.7(3)	0.010(4)	2.11(1)	0.4(4)	0.008
	Pd–In	5.5(2)	0.0094(4)	2.745(3)	1.3(4)	
	In–In	3.0(4)		3.220(9)	0.4(4)	

a Pd edge: S_0_^2^ = 0.80 from Pd metal; Δ*k* = 3.6–14.5 Å^–1^; Δ*R* = 2.0–3.0 Å. In edge: S_0_^2^ = 0.93 from In_2_O_3_; Δ*k* = 2.5–14.0 Å^–1^; Δ*R* = 1.2–3.2 Å.

**Figure 7:**
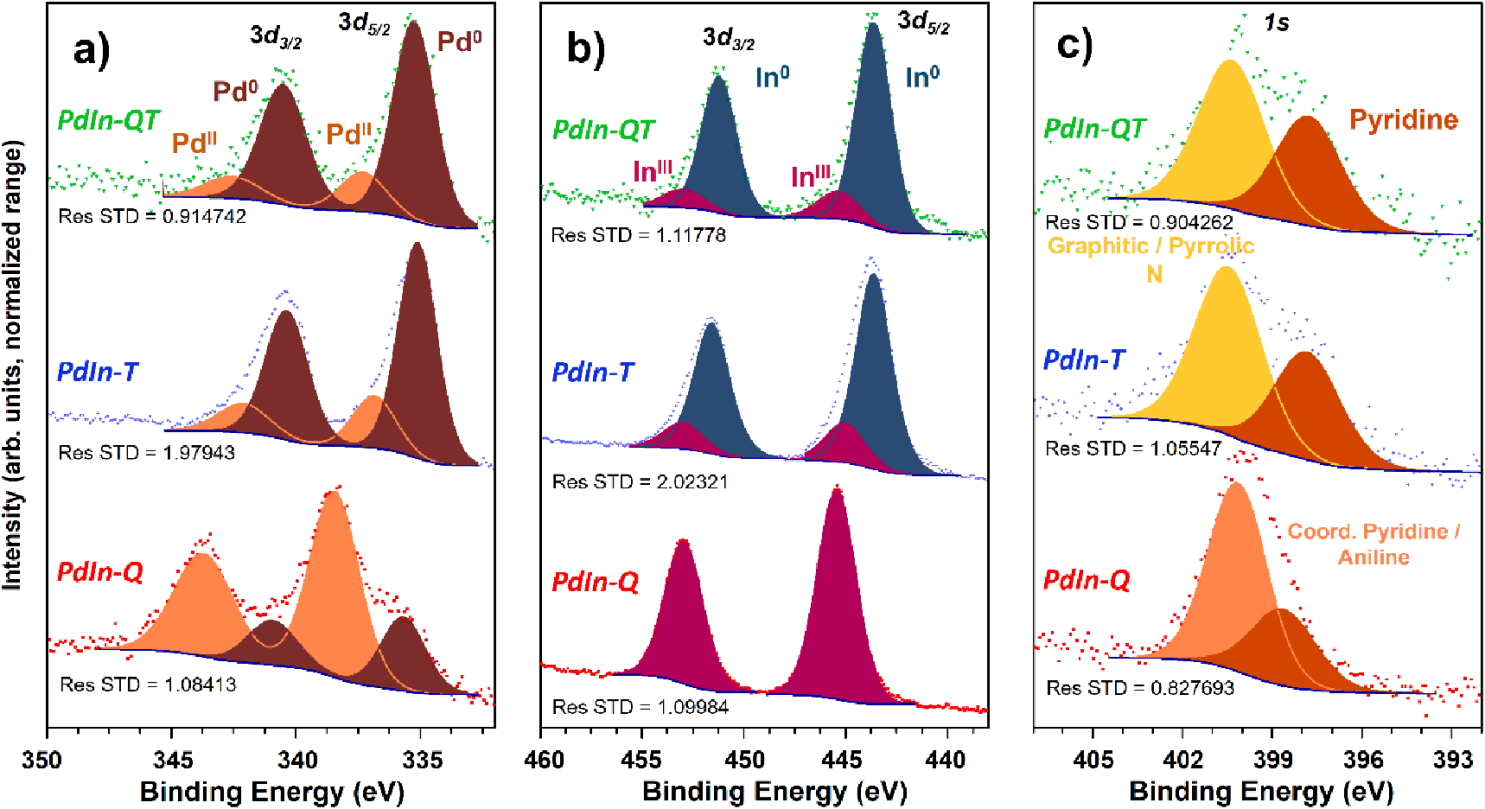
(a) Pd3*d*, (b) In3*d*, and c) N1s XPS regions of MOF-derived PdIn samples.

Additionally, the electronic state of the different elements composing the samples prepared in this work was analyzed by XPS. Figure 7 summarizes the most relevant information for the discussion herein, while the reader is referred to the SI (Figure S9 and Annex 1) for complete spectroscopic data. Regarding the two contributions in Pd*3d*_5/2_, the peak at higher B.E. is to be ascribed to surface oxidized Pd, positively shifted in **PdIn-Q**, likely implying coordination to Cl^–^ or the presence of Pd(IV).^60,61^ More interestingly, the peak at lower B.E. characteristic of Pd(0) in **PdIn-T** (335.1 eV), and **PdIn-QT** (335.3 eV), is shifted toward higher binding energies with respect to what is often reported for monometallic Pd.^60^ Contrarily, in these samples, the In3*d*_5/2_ peaks corresponding to In(0), arising after the thermal treatment, seems to shift toward lower binding energies (≈443.5 eV) relative to those of the elsewhere reported monometallic samples.^62^ These observations are consistent with a system with PdIn nanoparticles, as observed by HR-TEM, where these shifts might be explained in terms of a simple charge transfer model Pd^δ+^–In^δ−^.^63^ Moreover, this conclusion can be further reinforced by analyzing the Pd3*d* spectra of the Pd metalloligand treated by both the chemical and the thermal method, which, as expected for a monometallic system, shows the contribution of Pd(0) to Pd3*d*_5/2_ at 334.9 eV (Figure S9). Nonetheless, this upshift for Pd(0) is also seen in **PdIn-Q**. In fact, a similar effect can also be due to the interaction between In–O and Pd(0).^64^ Therefore, what can only unequivocally be assumed from XPS analyses is that Pd and In are closely interacting with each other in all these samples (**PdIn-Q, PdIn-T, PdIn-QT**). Therefore, the EM and XAS results are what distinguish the predominant interactions in each composite: Pd–In (in **PdIn-T** and **PdIn-QT**) and Pd–In–O (in **PdIn-Q**).

Concerning the N1s region (Figure 7c), the signal at 400.1 eV in **PdIn-Q** may be due to both the remnant pyridinic ligand coordinated to Pd^65^ and some aniline attached to the composite surface.^66^ On the other hand, the signal at a B.E. close to 398 eV, seen in all spectra in Figure 7c, may come from pyridinic nitrogen losing its coordination to Pd to different extents. Lastly, in the **PdIn-T** and **PdIn-QT** samples, a new band emerges at around 400.5 eV. This B.E. can tentatively be explained based on the appearance of a new type of nitrogen doping the graphitic carbonaceous support detected by Raman, in good agreement with the C–N–O component observed by STEM–EELS SI.^67,68^

### Catalytic Activity

**Figure 8:**
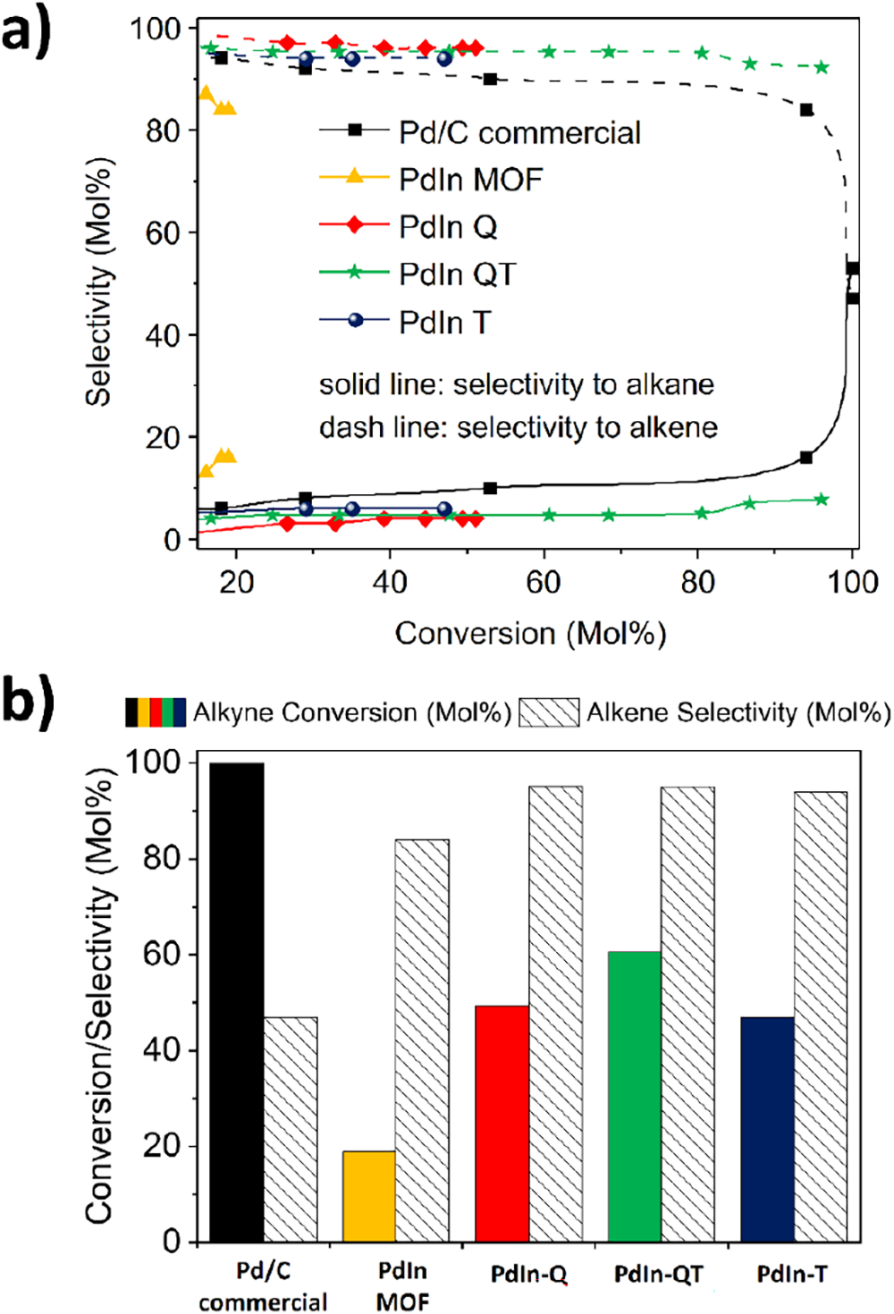
(a) Conversion vs selectivity plot of various PdIn-based materials used in the selective hydrogenation of phenylacetylene. (b) Activity and selectivity comparison of several PdIn-based materials after 6 h of phenylacetylene hydrogenation reaction. Reaction conditions: 5 mmol of phenylacetylene, substrate/Pd molar ratio: 323/1, 5 mL EtOH, R.T., 1 bar H_2_, 1000 rpm.

In Figure 8, catalytic performances of previously presented materials were evaluated and compared in the selective hydrogenation of phenylacetylene. According to the literature, catalysts based on Pd exhibit high activity in this reaction.^24^ However, their activity often results in over-reduction of alkenes, leading to a significant decrease in alkene selectivity.^6,7^ On the other hand, the use of Pd-M bimetallic catalysts (M: non-noble metal) has been widely employed to achieve better selectivity than corresponding monometallic Pd-based systems.^10−19^ Indeed, when palladium is close to another element like Indium or Gallium, the electron density of Palladium changes. This results in a decrease in alkene adsorption strength and an increase in alkene selectivity.^62,69,70^

In entry 1 of Table 3, the performance of the commercial Pd on the carbon catalyst has been investigated. As anticipated, the commercial catalyst shows a high activity level, resulting in over reduction and a low selectivity toward alkenes (as shown in Figure 8). Meanwhile, the activity of the newly depicted PdIn bimetallic materials has also been analyzed. Specifically, the **PdIn-QT** catalyst has proven to be a standout performer, selectively converting alkenes while maintaining significant alkyne conversion. Also, Figure S17 compares **PdIn-QT** and **Pd/C** commercial catalysts at conversion above 90%, highlighting the significance of Indium introduction to the catalyst in achieving high selectivities at high conversion levels. Remarkably, the very soft reaction conditions must be highlighted (room temperature, ethanol as solvent, and 1 bar of H_2_).

**Table 3:**
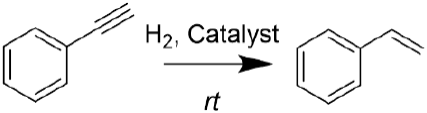
Comparison of Activity and Selectivity of Different Catalysts in Phenylacetylene Hydrogenation^a^

entry	catalyst	time (h)	conv. (mol %)	selec. (mol %)	TON	TOF (h^–1^)	productivity (g_alkyne_· g_cat_^–1^· h^–1^)
1	**Pd/C commercial**	7	100	47	322.0	46.0	2.1
2	PdIn-MOF	7	19	84	27.4	3.9	0.85
3	**PdIn-Q**	7	49	95	85.9	12.3	4.9
4	**PdIn-QT**	7	61	95	98.4	14.0	6.1
5	**PdIn-QT**	11	96	93	154.8	14.1	6.0
6	**PdIn-T**	7	47	95	67.1	6.1	4.7

a Reaction conditions: 5 mmol of phenylacetylene, substrate/Pd molar ratio: 323/1, 5 mL EtOH, R.T., 1 bar H_2_, 1000 rpm.

In entry 2 of Table 3, **PdIn-MOF** showed the lowest performance of all bimetallic tested materials (conversion 19% – selectivity 84%). This was expected as the material mostly contained oxidized palladium. Then, materials derived from chemical, thermal, and chemical–thermal treatments (**PdIn-Q, PdIn-T,** and **PdIn-QT,** respectively) were also studied. According to entries 3 and 6, **PdIn-Q** and **PdIn-T** have similar activities, with less than 50% conversion and 95% alkene selectivity after 7 h of reaction. Finally, the optimal catalytic performance was observed in the **PdIn-QT** material, which could achieve a conversion rate of 61% and a selectivity rate of 95% after 7 h of reaction time (entry 4). Even after 11 h of reaction time and a conversion rate of 96%, the material was able to maintain a selectivity of 93% (entry 5 and Figure S15). In order to understand the different behaviors of bimetallic materials derived from **PdIn-MOF**, there are a few crucial points to consider. While it has been previously shown that the **PdIn-Q** catalyst consists of monometallic nanoparticles, it is essential to note that Indium exists as highly isolated species associated with oxygen. In fact, the XPS analysis section revealed an interaction between Pd and In that could make the Pd site ideal for promoting high selectivity.^62,69,70^ However, it appears that the active sites of this material are not easily accessible to achieve a significant level of activity, probably due to aniline remaining on the surface (see Table 1 ICP/AE). Moreover, apart from the lower Pd accessibility, Figure S14a also shows the result of a hot filtration test and the corresponding ICP analysis conducted on the end reaction mixture. The analysis indicates that 1.4 wt % of Pd and 4.6 wt % of In have leached out of the total metal loading of the material.

As for the **PdIn-T** and **PdIn-QT** catalysts, according to the previous characterization discussion, it is clear these two materials have similar natures. Both are carbon supported PdIn bimetallic nanoparticles. Nonetheless, while both improve selectivity with respect to the commercial Pd/C (Table 3: Entry 1 and Figure S16), **PdIn-QT** is significantly more active than **PdIn-T** (Table 3: entries 4 and 6, Figure S14b,c). The difference between both catalysts could be attributed to several reasons. On the one hand, combining chemical and thermal treatments used to prepare PdIn-QT resulted in higher surface areas (175 m^2^·g^–1^; Table 1) and better control over nanoparticle size distribution (13 ± 6 nm). In comparison, **PdIn-T** had a surface area of only 104 m^2^·g^–1^ (Table 1) and a larger nanoparticle size (18 ± 10 nm). The increased surface area and narrower nanoparticle size distribution of **PdIn-QT** likely provide more active sites, which could explain its higher activity. On the other hand, the chemical treatment before pyrolysis introduces a small amount of aniline into the material, leading to enrichment in N. This phenomenon is confirmed by comparing elemental analysis of each material (Table 1: 2.9 wt % vs 1.4 wt %, for **PdIn-QT** and **PdIn-T**, respectively). According to the literature, nitrogen enrichment in the material can improve the hydrogen dissociation capability and stability.^71,72^

Following the previous findings, this study aimed to conduct a detailed analysis of the behavior of the **PdIn-QT** catalyst. Several experiments were carried out to demonstrate the versatile and selective nature of the **PdIn-QT** catalyst in the process of selective alkyne semihydrogenation.

First, the selectivity toward alkenes was experimentally demonstrated by comparing independent kinetic curves of phenylacetylene and styrene hydrogenation (Figure S14e). After 7 h of the reaction, the conversion of alkyne amounted to approximately 60%, while the conversion of alkene was only about 20%. As an estimate of the selectivity of our catalytic system, the average reaction rate of alkyne hydrogenation is three times higher than that of alkene. In order to confirm this affinity for alkynes, an experiment was performed using a mixture of alkynes and alkenes in a 1:9 ratio (Figure S14d). Even though the alkene concentration was nine times higher compared to that of alkyne, the catalyst **PdIn-QT** only converted the alkyne after 7 h of reaction time, whereas the alkene was barely converted. Then, the temperature effect on the catalyst performance was also studied. The reaction temperature was varied from 24 °C (r.t.) to 40 °C and up to 60 °C (Figure S14f). As expected, the higher the temperature, the higher the activity, but remarkably, the selectivity remained up to 95% regardless of the temperature, thus confirming the high selectivity of the catalyst composite herein designed.

To evaluate the degree of selectivity induced by the catalyst, we conducted a comparable set of experiments using a commercial Pd/C catalyst, as shown in Figure S16b. In accordance with the literature, this monometallic catalyst is highly active, but has relatively low selectivity compared to our system. Figure S16b compares the affinity of the Pd monometallic catalyst for double or triple-bond-based substrates. Interestingly, the reaction rate of alkyne hydrogenation is now almost equal to or even less than that of the alkene. After 7 h of reaction, alkyne and alkene substrates were converted to 99%. Consequently, when both substrates are present in the reaction medium (Figure S16c), the catalyst cannot maintain selectivity toward alkene compared to the **PdIn-QT** catalyst.

**Figure 9:**
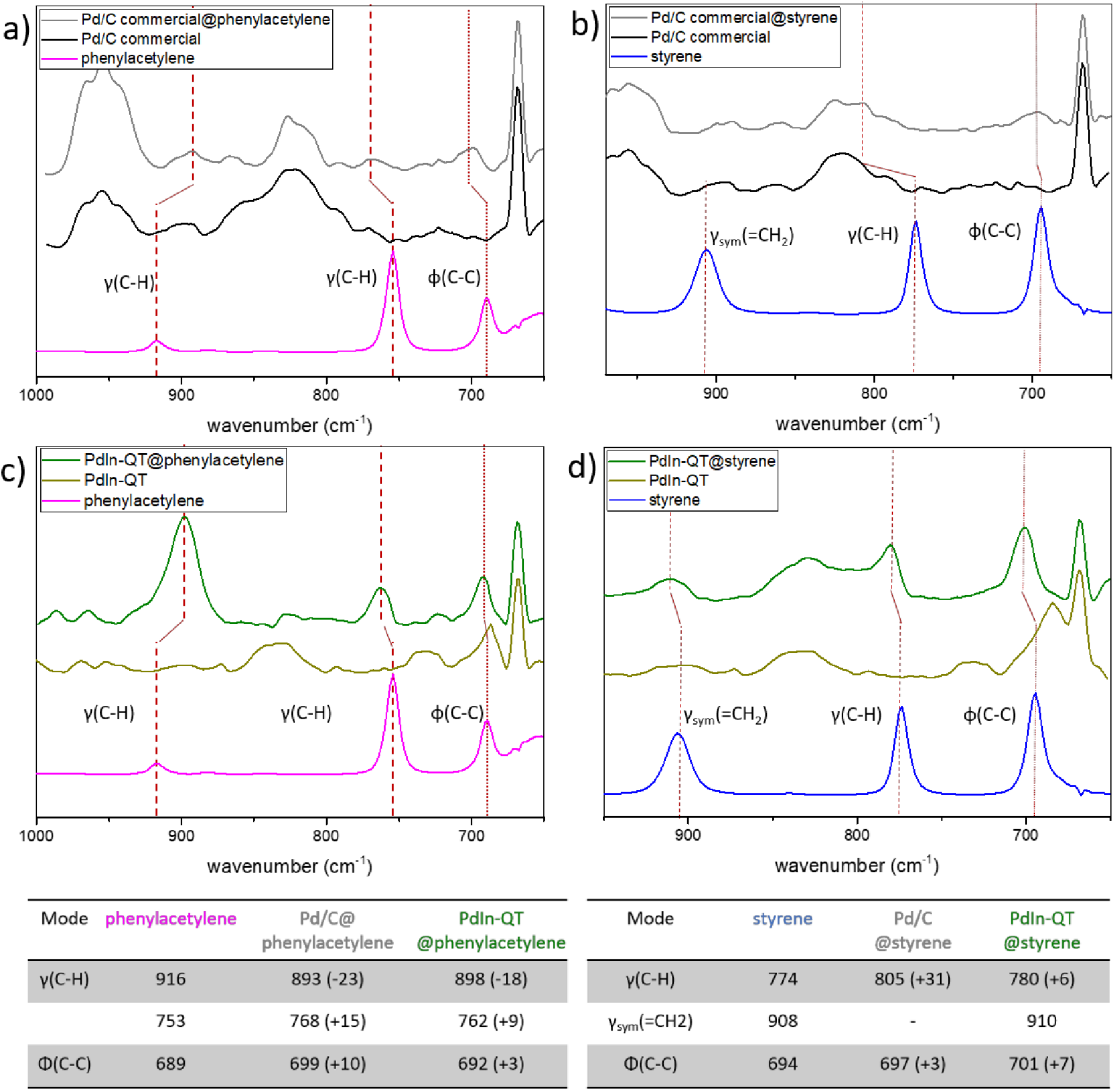
(a) FTIR spectra from bare Pd/C catalyst (black line), free phenylacetylene (purple), and phenylacetylene preadsorbed on Pd/C (grey). (b) FTIR spectra equivalent to (a), but with styrene (blue). (c) and (d) analogue FTIR spectra with the PdIn-QT catalyst (green) and preadsorbed molecules (dark green).

Therefore, monometallic (Pd/C commercial) and bimetallic (**PdIn-QT**) materials showed two different catalytic behaviors, likely implying preferential superficial substrate adsorptions onto the catalyst. In this sense, Figure 9 summarizes in situ DRIFT spectroscopy carried out at room temperature. First, free molecule spectra of styrene and phenylacetylene were recorded, and the IR absorption band attribution was performed thanks to a previously reported work from Liu and coworkers.^73^ Despite the non-transparency of the carbon support, spectra of free catalysts and adsorbed probe molecules onto catalysts could be acquired. On the one hand, the shifts toward the upper or lower energy of the adsorbed molecules band compared to the free molecules reflect a strong interaction with the catalysts. On the other hand, the difference in adsorption between the two materials should be highlighted. In the case of the Pd/C commercial catalyst, the IR-absorption bands of phenylacetylene and styrene in the adsorbed phase are impacted. Specifically, the γ(C–H) bands are significantly affected in the case of the adsorbed triple bond molecule (−23 cm^–1^, + 15 cm^–1^), but even more so in the case of the adsorbed double bond molecule (+31 cm^–1^). However, γ(C–H) bands are less affected in the case of the **PdIn-QT** material. Additionally, there seems to be a stronger interaction between the catalyst and phenylacetylene than with styrene. In that sense, the γ(C–H) band presents a relatively low shift (+6 cm^–1^) for the styrene compared to the adsorbed bands of phenylacetylene (−18 cm^–1^, + 9 cm^–1^). In conclusion, the Pd/C commercial catalyst shows greater interaction with the substrates compared to the **PdIn-QT** catalyst. More specifically, the Pd/C commercial catalyst has a slightly stronger affinity toward styrene, which could result in over-reduction and a loss of previously demonstrated catalytic selectivity. Therefore, it can be postulated that the higher catalytic activity, but lower selectivity of the Pd/C commercial catalyst is due to a strong interaction with both, alkene and alkyne-type substrates. Remarkably, this is in good agreement with what the literature has reported regarding the role of In in semihydrogenation catalysis when it is combined with Pd. In that sense, Q. Feng et al. calculated the desorption energy of C_2_H4 on one Pd atom in the Pd_1_In_1_(110) surface via a weak π-bond to be lower than its hydrogenation barrier, and the transition-state energy of C_2_H_4_ hydrogenation to be above the energy of gas-phase ethylene, which suggests that ethylene prefers desorption to further hydrogenation.^13^ Finally, the **PdIn-QT** catalyst has weaker adsorbed band perturbations and a stronger interaction with phenylacetylene with respect to styrene. This fact should certainly be a driving force behind maintaining the selectivity of the process toward alkene production.

Table 4 presents the results of the semihydrogenation reactions using the **PdIn-QT** catalyst with different alkynes under similar reaction conditions. The corresponding kinetic curves of each reaction depicted in Table 4 are shown in Figures S14 and S18. The **PdIn-QT** catalyst exhibits high activity and maintains considerable selectivity across various substrates. Notably, it demonstrates enhanced reactivity toward aromatic substrates compared to aliphatic ones (Table 4: entries 1 and 8). This behavior is attributed to the carbon support’s ability to establish effective π–π stacking interactions with aromatic substrates.^74,75^ Also, the meta- or para-position of a functional group in an aromatic alkyne affects the reactivity. Comparing entries 6 and 7 of Table 4, an alkyne substrate with a functional group in meta-position is more reactive than a substrate with a functional group in para-position. According to the literature, the electron density in the alkyne group is strongly affected by a functional group in the para-position of the aromatic substrate due to electron density movement through the conjugated molecules.^75,76^ The reactivity of the **PdIn-QT** catalyst is significantly influenced by the nature of functional groups. Substrates with electron-withdrawing groups (EWG), like nitro or carboxylic groups in the para position, exhibit higher activity in activating the alkyne triple bond compared to substrates with electron-donor groups (EDG), such as amines, which deactivate the alkyne group (Table 4: entries 4 and 5). This trend is attributed to the EWG inducing a partial δ+ charge on the alkyne group, facilitating better coordination with the active Pd sites in our catalyst.^77^ On the other hand, the presence of hydrogen bonded to electronegative heteroatoms (N or O) in functional groups facilitates H interactions with the active site, impacting catalytic activity. Notably, substrates with −COOH and −NH_2_ functional groups (Table 4: entries 3, 4, and 5) exhibit longer reaction times compared to substrates without H-bonded functional groups like halogens (Table 4: entry 6). This suggests a competitive adsorption scenario between functional groups in the substrate.

**Table 4:**
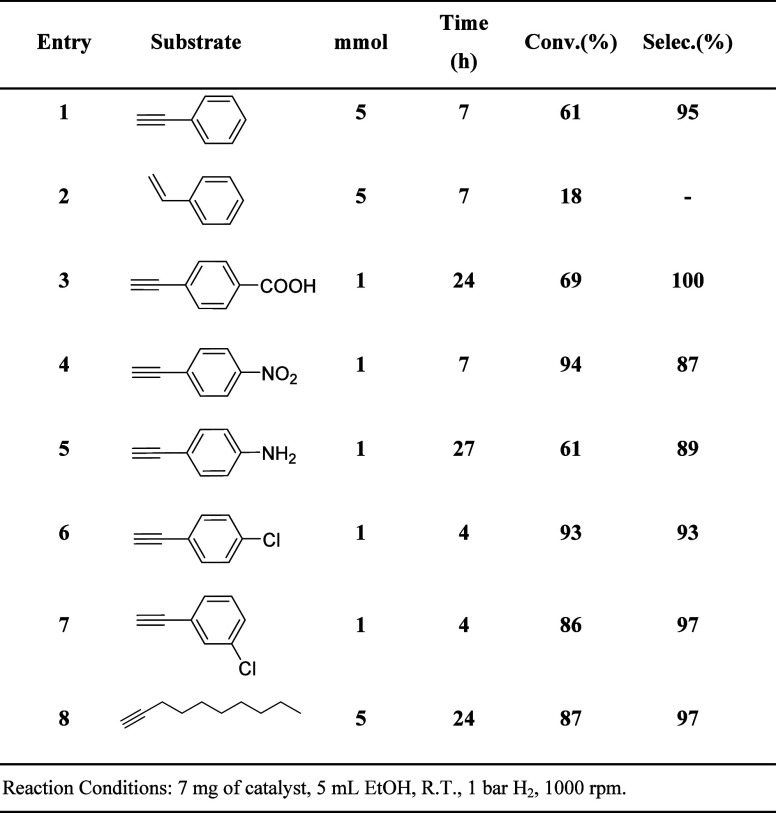
Hydrogenation Reaction Results of Various Substrates Over the **PdIn-QT** Catalyst

### Catalytic Stability

**Figure 10:**
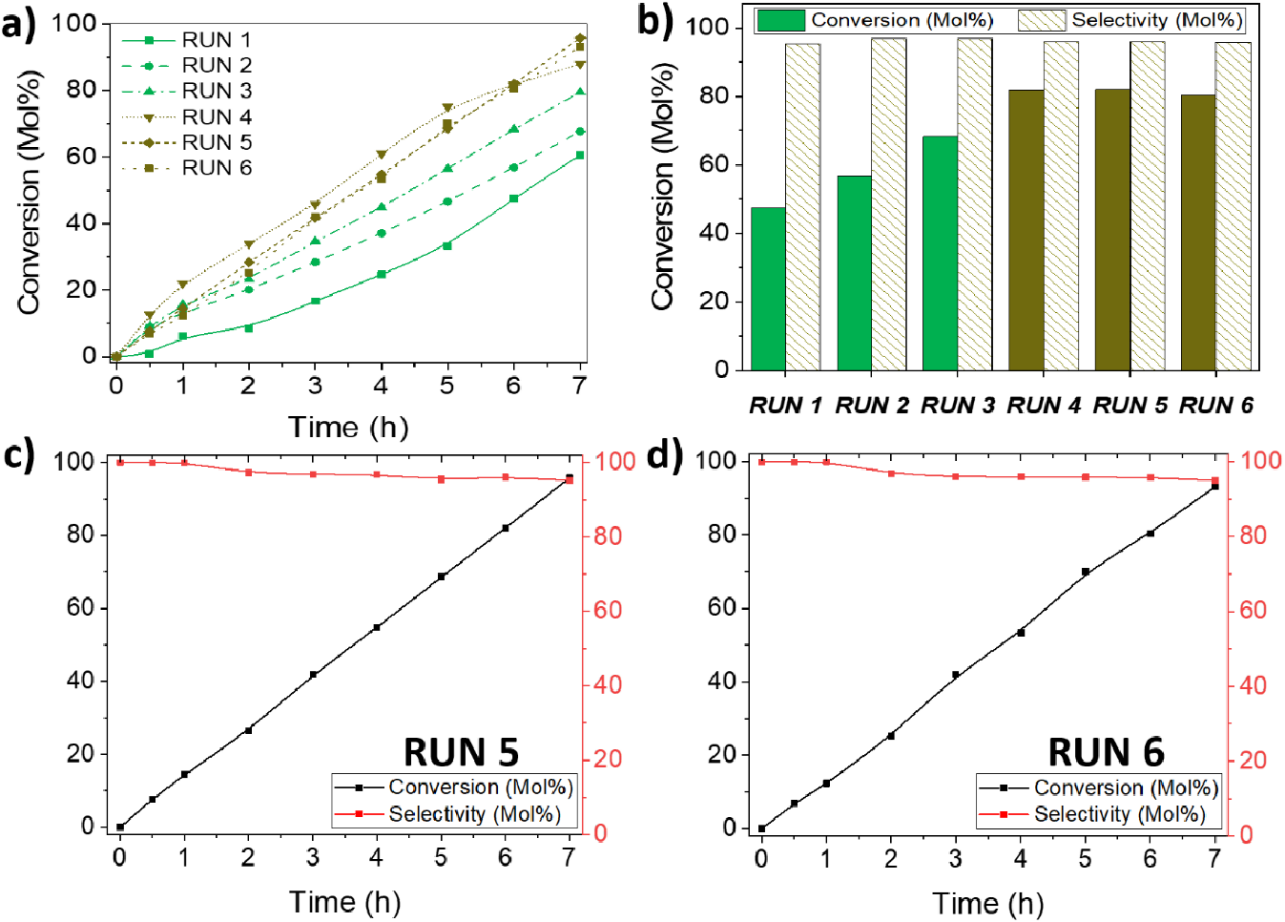
(a) kinetic curves of **PdIn-QT** catalyst during runs 1 to 5, (b) stability cyclic test of the **PdIn-QT** catalyst at 6 h reaction time, (c) and (d) kinetic curves of the **PdIn-QT** catalyst during runs 5 and 6. Reaction conditions: 5 mmol of phenylacetylene, substrate/Pd molar ratio: 323/1, 5 mL EtOH, R.T., 1 bar H_2_, 1000 rpm.

In Figure 10, we depict the stability study of our catalytic process. It is noteworthy that our catalyst activity shows an increasing trend until run 4, where it stabilizes. Interestingly, this stabilization does not seem to affect selectivity. Eventually, after four 7 h long runs (Figures 9c,d and S19), 7 mg of **PdIn-QT** catalyst can convert 5 mmol of phenylacetylene at 96% and be selective to styrene at 96% with only 1 bar of H_2_ in ethanol, which translates into TON = 150 and TOF = 21.4 h^–1^ values. It is important to note that in comparison to other Pd-based catalysts, the **PdIn-QT** catalyst under consideration has a high productivity rate of 9.4 (g_alkyne_ · g_cat_^–1^ · h^–1^). This stands out as one of the best-reported Pd-based catalyst values under similar reaction conditions in our study, as indicated in Table S4.

**Figure 11:**
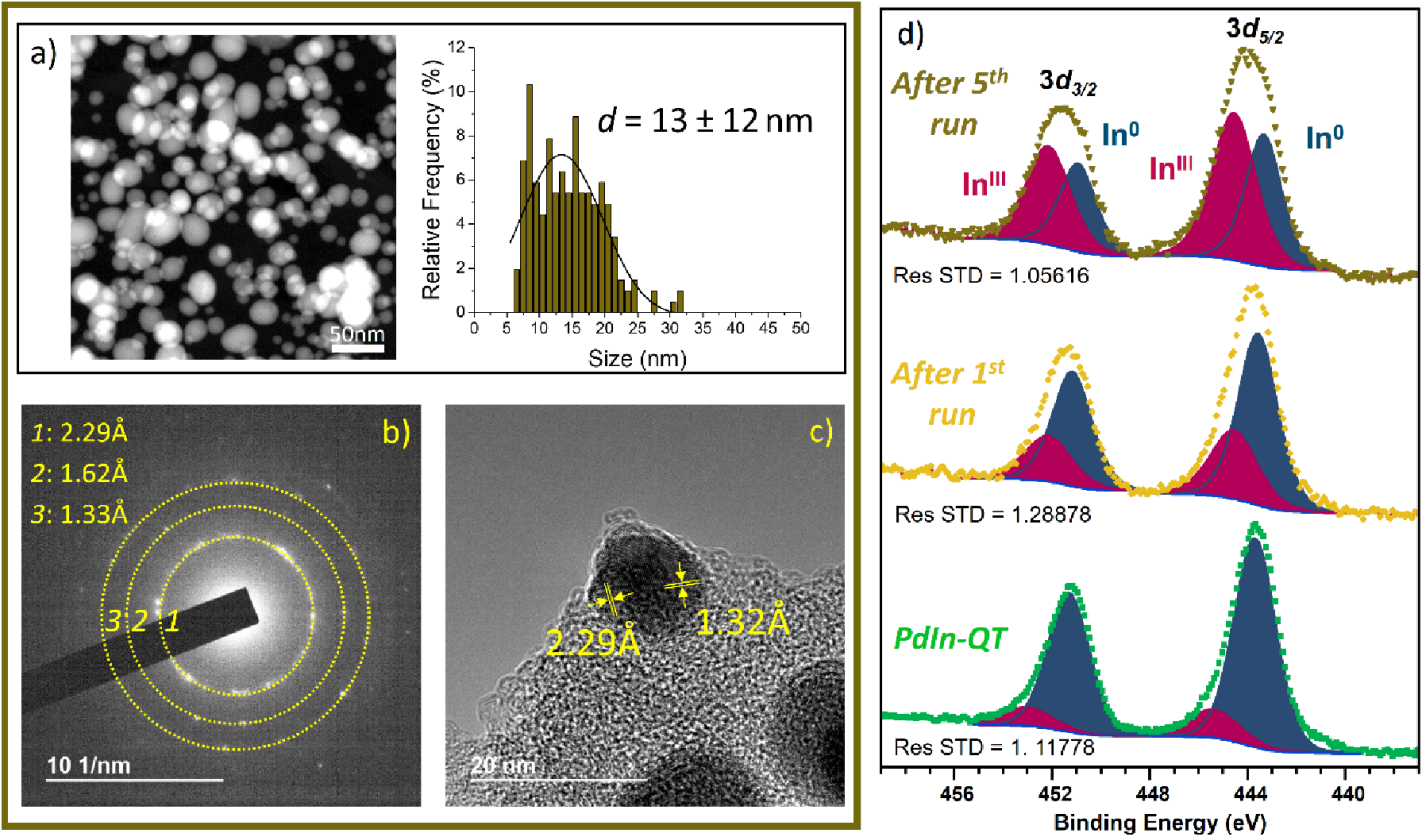
Microscopy results for **PdIn-QT** after 5 catalytic cycles. (a) HAADF–STEM image and corresponding nanoparticle size distribution, (b) SAED patterns, (c) HRTEM image with measured interplanar distances, and (d) In3*d* XPS regions of **PdIn-QT** fresh, after 1st run and 5th run.

To explain the upward trend behavior observed over consecutive uses, the **PdIn-QT** catalyst after run 5 has been extensively studied by XRD, XPS, HRTEM, EDX, EELS, and Raman (Figures 11 and S20–S23). The catalyst showed no noticeable changes in its metal distribution, X-ray diffraction pattern, and Raman spectrum postreaction, closely resembling its original state, except for a slight broadening of the nanoparticle size distribution. Therefore, the **PdIn-QT** catalyst appears as a very stable material with a successful anchoring of the PdIn NPs on the carbon support. However, after the fifth run, the outer C–O layer around the nanoparticles disappeared, as confirmed by EELS analysis. This fact suggests a better accessibility of the substrate to the active site that could account for its higher activity throughout the stability study. In the same line, XPS revealed the occurrence of surface oxidation on Indium (Figure 11d). It has been claimed that oxygen vacancies created by this partial oxidation could also contribute to the increasing activity, via an enhancement of the H_2_ dissociation step.^78^

## Conclusions

In our research, we explored several procedures, including thermal, chemical, and chemical–thermal methods, to transform a **PdIn-MOF** into a carbon-supported metallic nanoparticle system. The goal was to enhance the selectivity of conventional Pd-based catalysts for alkyne semihydrogenation reactions. Advanced structural analyses using XRD, HR-EM, Raman, XAS, and XPS have demonstrated that the thermal (pyrolytic) method promotes stability and enhances the bimetallic character of the metal nanoparticles. Conversely, the soft chemical treatment yielded smaller particle size distributions, preserved the high surface area of the parent MOF, and increased the nitrogen content of the final composite. Significantly, by combining both chemical and thermal routes, we synthesized a material (**PdIn-QT**) featuring well-defined PdIn bimetallic nanoparticles, high surface area, and robust stability. Consequently, this newly developed composite proved to be a highly effective catalyst for the hydrogenation of phenylacetylene to styrene, achieving a 96% conversion of phenylacetylene with a 93% selectivity to styrene within 11 h under moderate conditions. While this approach initially resulted in reduced catalyst activity, repeated use demonstrated a gradual improvement in the exposure of active sites, reaching a plateau after the third use with a maximum activity of 96% conversion in 7 h. At the same time, it maintains a 96% selectivity to styrene, resulting in a high productivity of 9.4 g_alkyne_·g_cat_^–1^·h^–1^, which remains consistent after further use. These findings not only introduce a novel catalyst for alkyne semihydrogenation but also highlight the potential to transform MOFs into nanoparticles through combined methodologies, yielding materials with enhanced structural properties and tailored catalytic activities.

## Supporting Information Available

Supporting Information includes a description of materials used and general experimental methods, characterization studies (^1^H NMR, TEM, and XPS) on the **PdIn-MOF** and **PdIn-Q** materials, additional XAS data, a summary providing XPS assignments, and complementary tests to evaluate the catalytic activity and the catalytic stability of the materials and literature reference data (PDF)
