# Supplementary material for: Efficient Alkyne Semihydrogenation Catalysis Enabled by Synergistic Chemical and Thermal Modifications of a PdIn MOF
Source: ACS Catal. 2024 Mar 14;14(7):4768–85. doi: 10.1021/acscatal.4c00310 (PMC11002973; doi:10.1021/acscatal.4c00310)
Supplement: Supplementary file 1 — cs4c00310_si_001.pdf [file cs4c00310_si_001.pdf]

Efficient Alkyne Semihydrogenation Catalysis Enabled by Synergistic Chemical and Thermal Modifications of a PdIn MOF

Jordan Santiago Martinez,[1] Jaime Mazarío,[2] Christian Wittee Lopes,[3] Susana Trasobares,[4,5] José Juan Calvino Gamez,[4,5] Giovanni Agostini[6] and Pascual Oña Burgos[1*]

*Corresponding author: pasoabur@itq.upv.es.

[1] *Instituto de Tecnología Química, Universitat Politècnica de València-Consejo Superior de Investigaciones Científicas (UPV-CSIC), Avda. de los Naranjos s/n, 46022 Valencia, Spain.*

[2] *LPCNO (Laboratoire de Physique et Chimie des Nano-Objets), Université de Toulouse, CNRS, INSA, UPS, 31077 Toulouse, France.*

[3] *Department of Chemistry, Federal University of Paraná (UFPR), Curitiba, 81531-990, Brazil.*

[4] *División de Microscopía Electrónica de los Servicios Centralizados de Investigación Científica y Tecnológica de la Universidad de Cádiz (DME-UCA), Facultad de Ciencias, Universidad de Cádiz, Campus Río San Pedro S/N, Puerto Real 11510, Cádiz, Spain.*

[5] *Departamento de Ciencia de los Materiales e Ingeniería Metalúrgica y Química Inorgánica, Facultad de Ciencias, Universidad de Cádiz, Campus Río San Pedro S/N, Puerto Real 11510, Cádiz, Spain.*

[6] *ALBA Synchrotron Light Facility, Carrer de la Llum 2-26, Cerdanyola del Valles, Barcelona 08290, Spain.*

Supporting Information

S1. Materials and General methods 2

S2. Synthesis and characterization...2

S2.1. Palladium complex (H_4L) and derived materials...2

S2.2. PdIn MOF..3

S2.3 PdIn derived materials...5

S3. Catalytic activity..12

S4. Catalytic stability..16

ANNEX 1. Summary of XPS assignments...19

References...20

S1. Materials and General methods

Materials: All reagents and solvents used here were of high purity grade and purchased from Merck company. Only, 4-ethynylaniline, 1-chloro-4-ethynylbenzene and 1-chloro-3-ethynylbenzene were purchased from TCI company.

S2. Synthesis and characterization

S2.1. Palladium complex ($\mathbf{H_4L}$*) and derived materials.*[1]

S2.1.1. Palladium complex synthesis ($\mathbf{H_4L}$)

Pd-based metal-ligand was prepared by the ligand exchange process. 1 mmol of bis(benzonitrile)palladium(II) chloride $(C_6H_5CN)_2PdCl_2$ and 2 mmol of pyridine-3,5-dicarboxylic acid were dissolved in 150 mL dry THF under inert atmosphere (Ar). Then, the mixture was stirred for 5 h at room temperature to yield a yellow solution. Then, the resulting mixture was concentrated, and the final product was precipitated using 250 mL of hexane. The product was collected by vacuum filtration, washed with THF/hexane and hexane and finally vacuum dried. The purity of the resulting metal complex has been checked by NMR ^1H in CD_3OD (Figure S1).

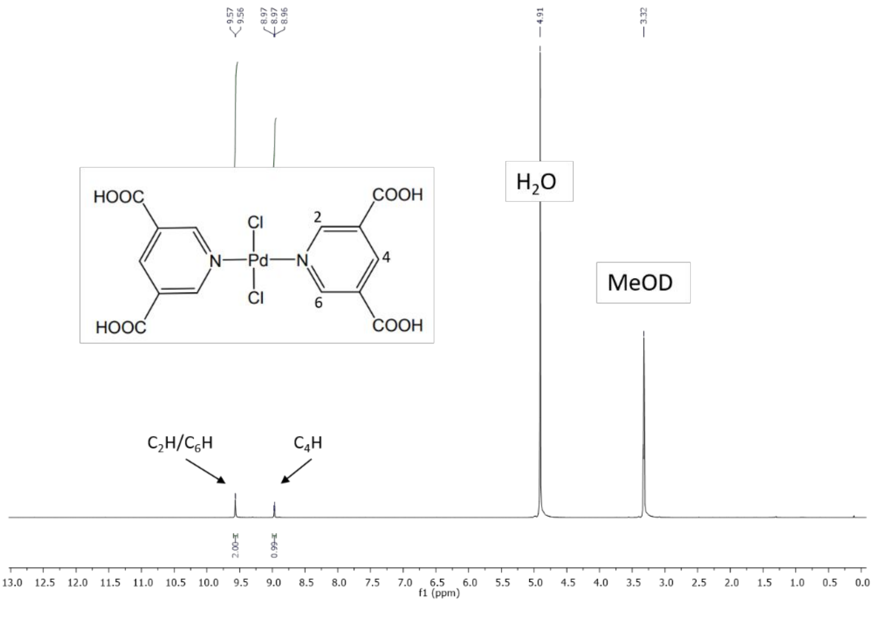

Figure S1. ^1H NMR spectra of synthesized Pd complex

S2.1.2. Preparation of **H_4L-T**

200 mg of **H_4L** was transformed by direct pyrolysis in a tubular fixed-bed reactor under N_2 flow (20 mL.min^{-1}) at 800 °C for 2 h (ramp 25 °C·min^{-1}). Then, the material was cooled down to room temperature under a higher N_2 flow (40 mL·min^{-1}).

S2.1.3. Preparation of **H_4L-Q**

H_4L (400 mg) were placed into a 300 mL hydrogenation reactor with a solution of 80 mmol of nitrobenzene and 80 mL of toluene (yellow mixture). The system was sealed and pressurized at 5 H_2 bar at room temperature. After 24 h of vigorous magnetic stirring, the resulting dark solution was filtrated under vacuum to recover the material. Then, the material was washed several times with methanol and activated at 300 °C under vacuum for 6 h.

S2.1.4. Preparation and characterization of **H_4L-QT**

In order to synthesize **H_4L-QT**, a pyrolytic thermal treatment was applied to the previously depicted material (**H_4L-Q**). Accordingly, 200 mg of PdIn-Q (before the activation step) were pyrolyzed in a tubular fixed-bed reactor under N_2 flow (20 mL·min^{-1}) at 800 °C for 2h (ramp 25 °C·min^{-1}). Then, the material was cooled down to room temperature with a higher N_2 flow (40 mL·min^{-1}).

2.2. PdIn-MOF

See Main Text (*Experimental* Section) for detailed synthesis procedure.

Table S1. ICP and EA results for **PdIn-MOF** sample.

Material	**Pd wt%[a]**	**In wt%[a]**	**N wt%[b]**	**C wt%[b]**	**H wt%[b]**
PdIn MOF	9.3	18.5	5.1	22.0	2.6

[a] From ICP. [b] From EA.

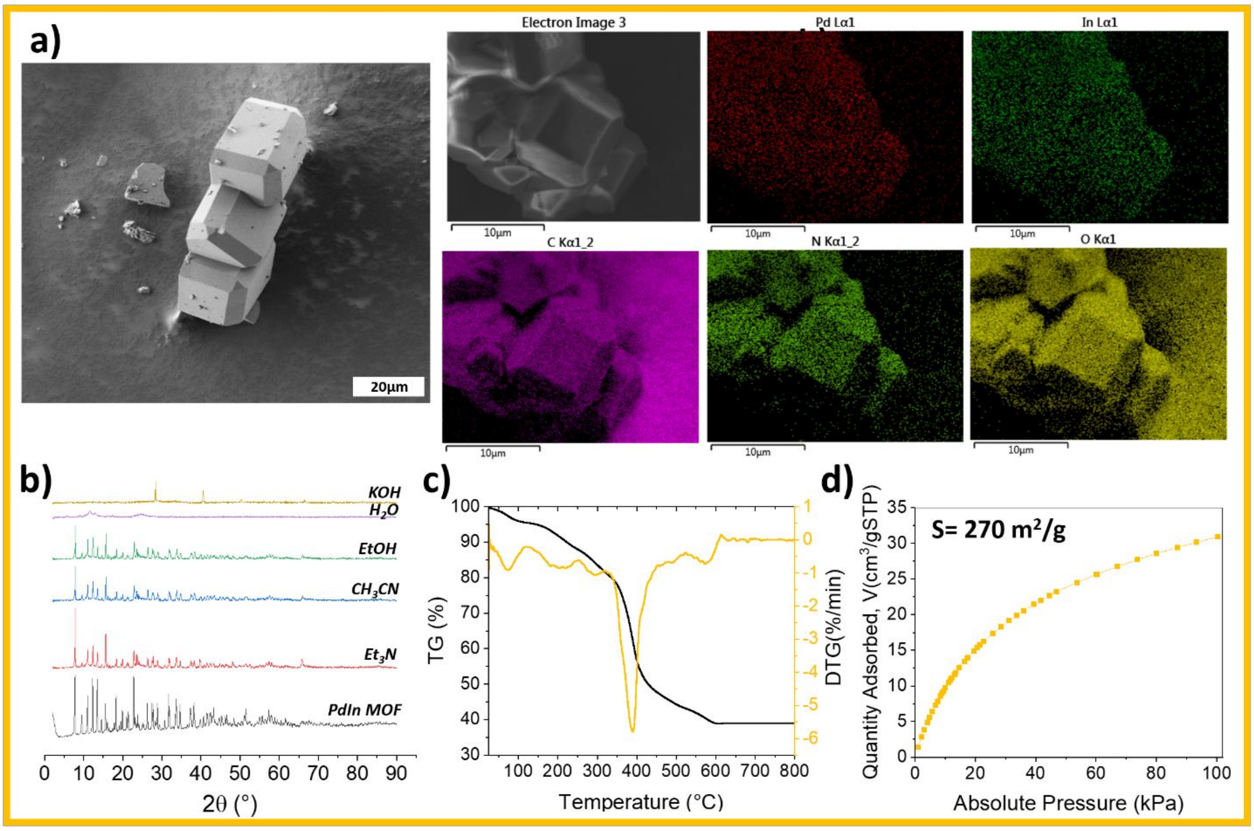

Figure S2. PdIn-MOF characterization. (a) SEM picture showing nanocrystals and the corresponding EDS analysis of Pd, In, C, N and O elements. (b) PXRD after immersion in different solutions during 24h (c) **Black**: Thermogravimetric analysis (TGA) using a heating rate of 25 $^{0}C\cdot min^{-1}$ under air flow. **Mustard**: The derivative of weight loss with temperature. (d) CO_2 gas adsorption isotherm measured at 273K.

S2.3. PdIn derived materials

See Main Text (*Experimental* Section) for complete synthesis protocols.

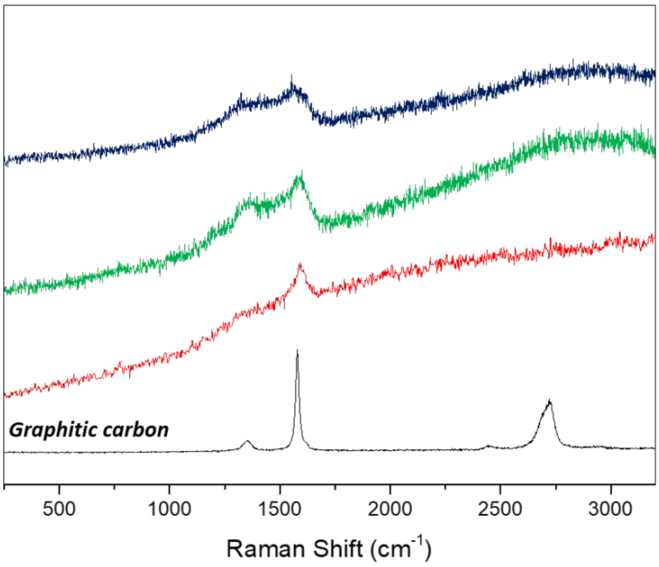

Figure S3. Raman spectra of PdIn-Q (**red**), PdIn-T (**blue**) and PdIn-QT (**green**) samples. I_D/I_G= 0.37, I_D/I_G= 0.74, I_D/I_G= 0.75.

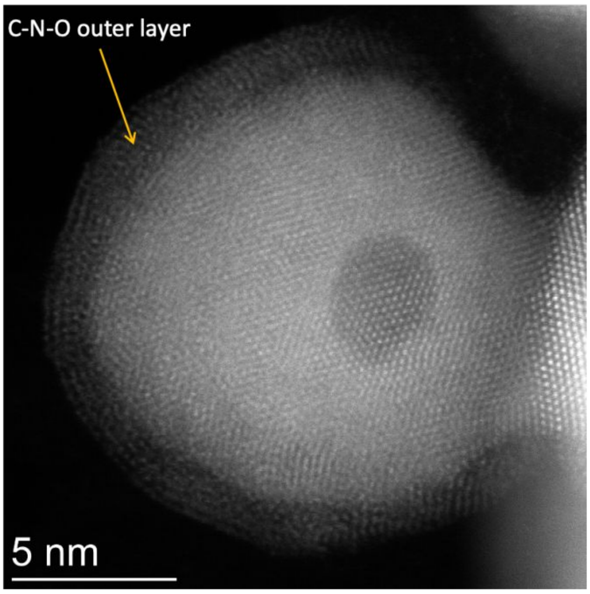

Figure S4. HR-HAADF STEM image of the **PdIn-T** catalyst showing the presence of an outer layer surrounding the intermetallic nanoparticle. Note the lower intensity of this layer, as expected from its content in light elements (C, N, O) as compared to those in the core (Pd, In).

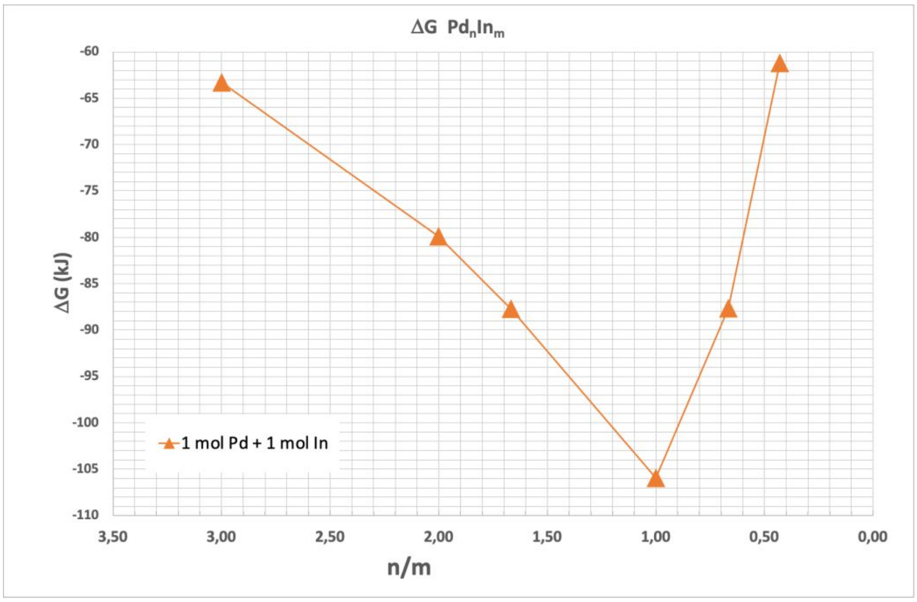

Figure S5. Total Free Energy Evolution during the reaction nPd + mIn → Pd_nIn_m considering an availability of reactants corresponding to a Pd/In molar ratio value of 1. The whole range of Pd-In stoichiometries are considered.

Table S2. Free energy of formation of the different Pd-In stoichiometries (data extracted from https://next-gen.materialsproject.org/materials/mp-21215?chemsys=Pd-In#thermodynamic_stability)[2]

**Pd-In stoichiometries**	**(ΔG_f^0 (kJ/mol)**
Pd_3In	-189.9
Pd_2In	-159.8
Pd_5In_3	-438.4
PdIn	-105.9
Pd_2In_3	-262.9
Pd_3In_7	-428.4

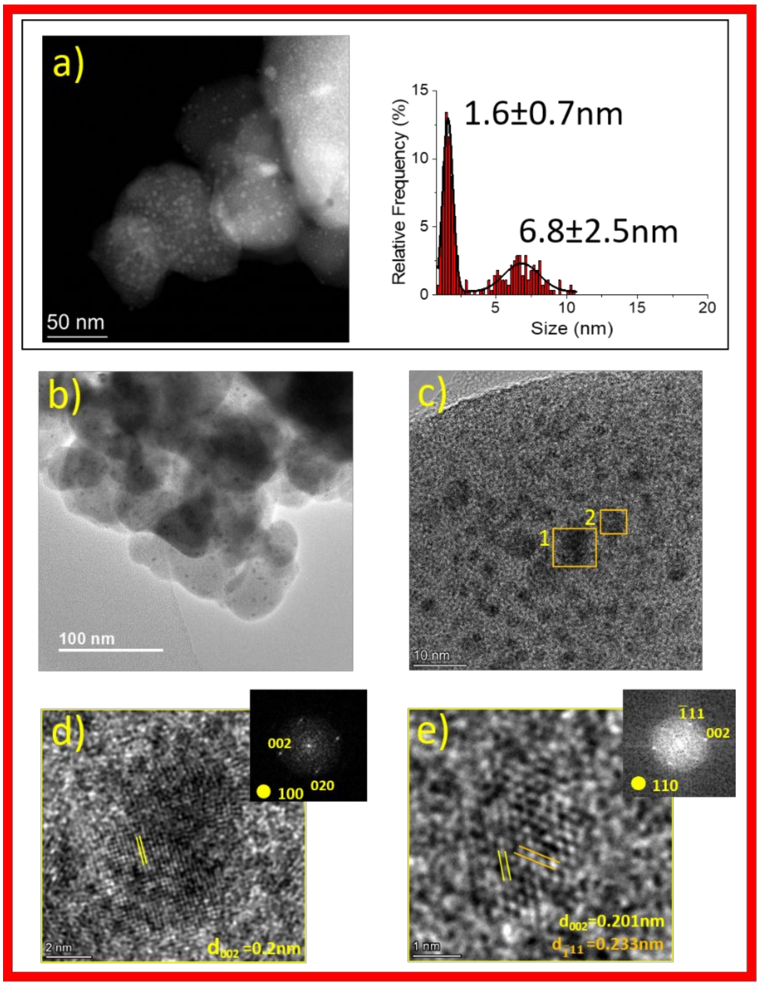

Figure S6. PdIn-Q material microscopy results, a) STEM-HAADF image and nanoparticle size distribution, b) and c) Representative HR-TEM image of the **PdIn-Q** catalyst. d) and e) HR-TEM images with measured interplanar distances.

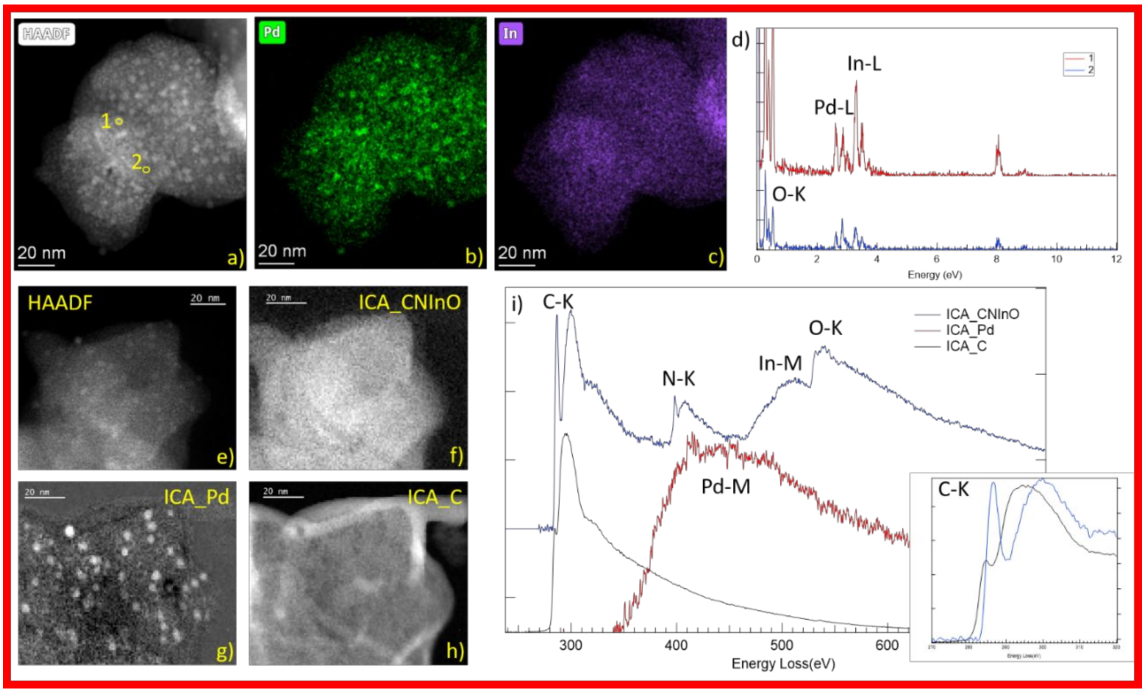

Figure S7. Results of the STEM-EDX and STEM-EELS of **PdIn-Q** sample a) HAADF image and the corresponding chemical maps Pd b) and In c), and d) a representative EDS spectra. STEM-EELS study including a HAADF image e) and the images corresponding to the three components of the ICA analysis of the whole set of STEM-EELS-SI data f), g) and h). EELS spectra corresponding to the three independent components i).

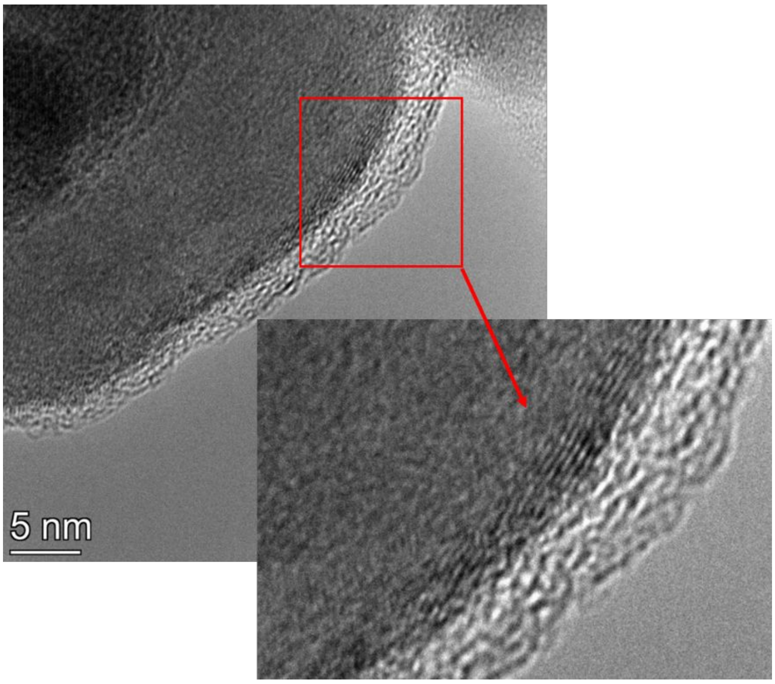

Figure S8. HRTEM image with zoom of carbon coated nanoparticle of **PdIn-QT** material. Both C-N-O and C-O layers are detected.

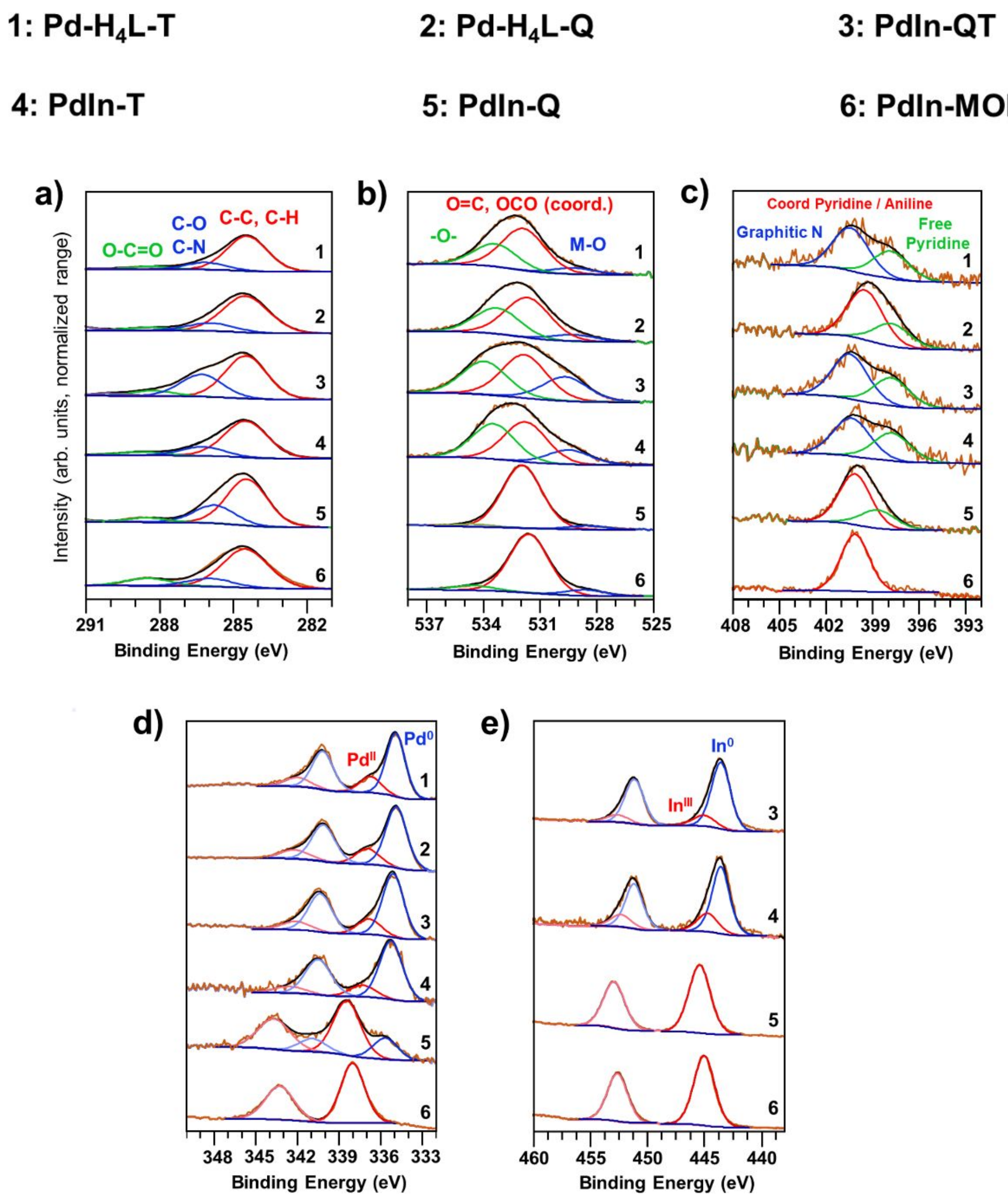

Figure S9. a) C*1s*, b) O*1s*, c) N*1s*, d) Pd*3d*, e) In*3d*, XPS regions of Pd-H_4L and MOF-derived PdIn samples.

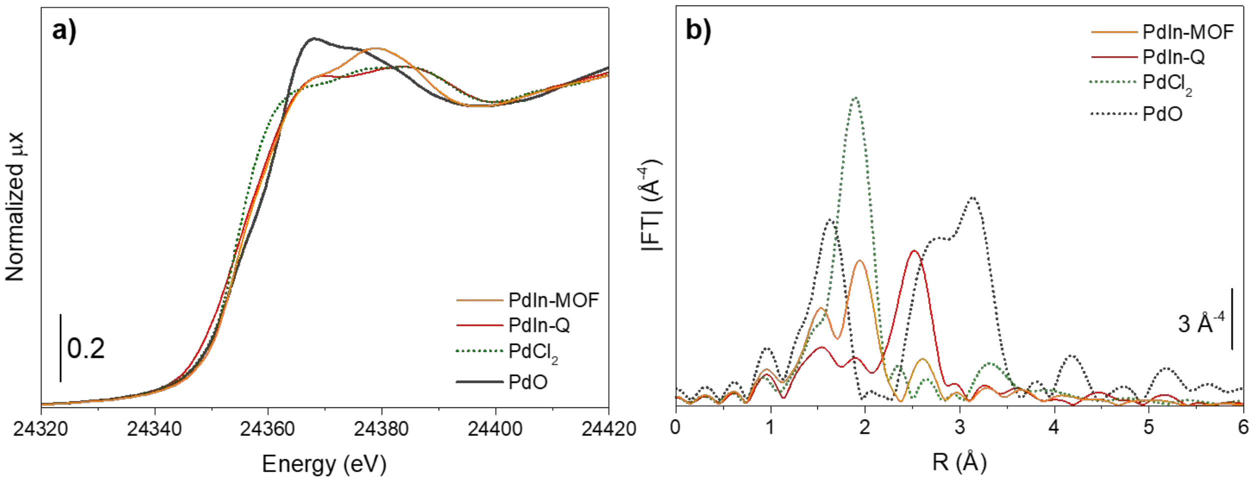

Figure S10. XANES spectra at Pd K-edge (a) and k^3-weighted |FT| EXAFS spectra (b) of PdIn-MOF, PdIn-Q and Pd-based references.

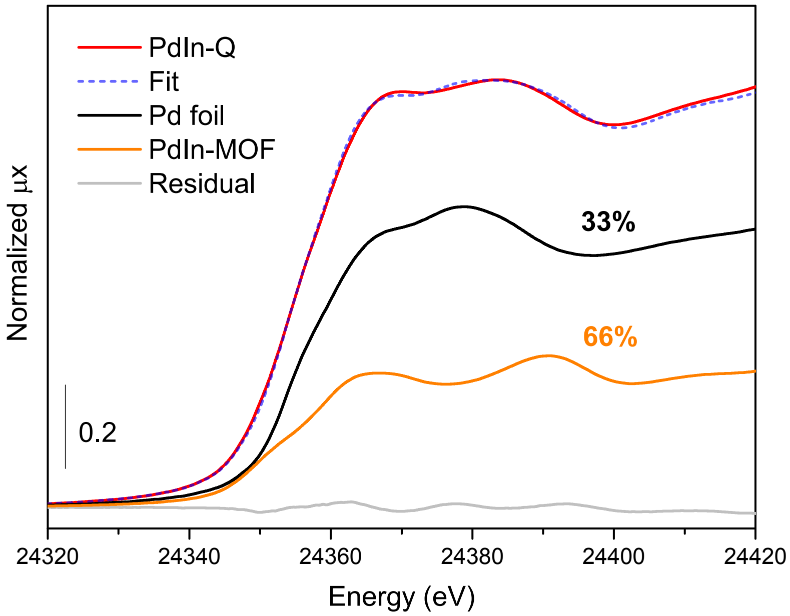

Figure S11. Linear combination fitting of PdIn-Q sample using Pd foil and PdIn-MOF spectra as references. No constrains have been applied to the fit.

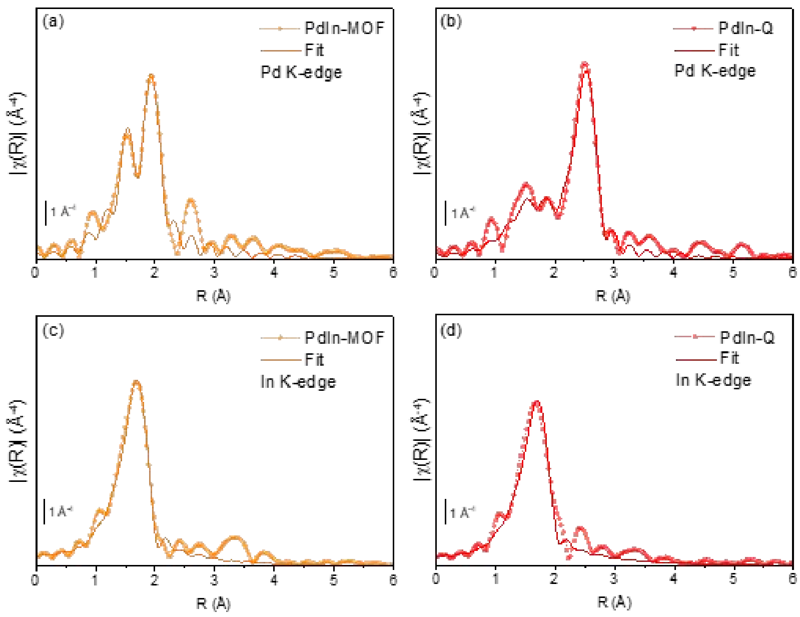

Figure S12. Curve-fittings and |FT| of the k^3-weighted χ(k) functions of PdIn-MOF and PdIn-Q at Pd and In K-edges. Coloured circles refer to experimental data while solid lines represent the fits.

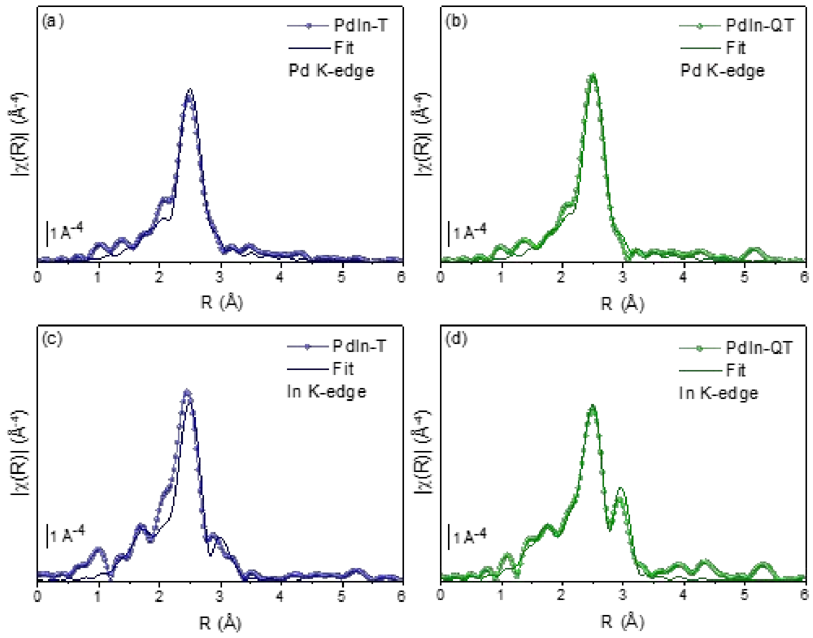

Figure S13. Curve-fittings and |FT| of the k^3-weighted χ(k) functions of PdIn-T and PdIn-QT at Pd and In K-edges. Coloured circles refer to experimental data while solid lines represent the fits.

Table S3. Summary of EXAFS fits of PdIn-MOF and PdIn-Q samples.

Sample	Path	N	Debye-Waller (Å^2)	R (Å)	ΔE (eV)	R-factor
PdIn-MOF	Pd-L	1.8(3)	0.002(1)	1.99(2)	5.5(1.7)	0.0055
	Pd-Cl	1.8(3)		2.307(2)		
	In-O	7.0(3)	0.0078(7)	2.159(5)	3.6(6)	
PdIn-Q	Pd-L	2.0(8)	0.011(3)	1.99[a]	0.8(7)	0.0181
	Pd-Cl	1.2(3)		2.307[a]		
	Pd-Pd	4.2(7)	0.007(1)	2.772(7)		
	In-O	6.7(8)	0.008(2)	2.169(13)	4.6(1.4)	

Pd edge: $S_0^2 = 0.80$ from Pd metal; Δk=3.6-14.5 Å^{-1}; ΔR=1.0-2.2 Å for sample PdIn-MOF, ΔR=1.0-3.0 Å for sample PdIn-Q. In edge: $S_0^2 = 0.93$ from In_2O_3; Δk=3.0-14.0 Å^{-1}; ΔR=1.1-2.0 Å. [a] fix from fit on PdIn-MOF

S3. Catalytic activity

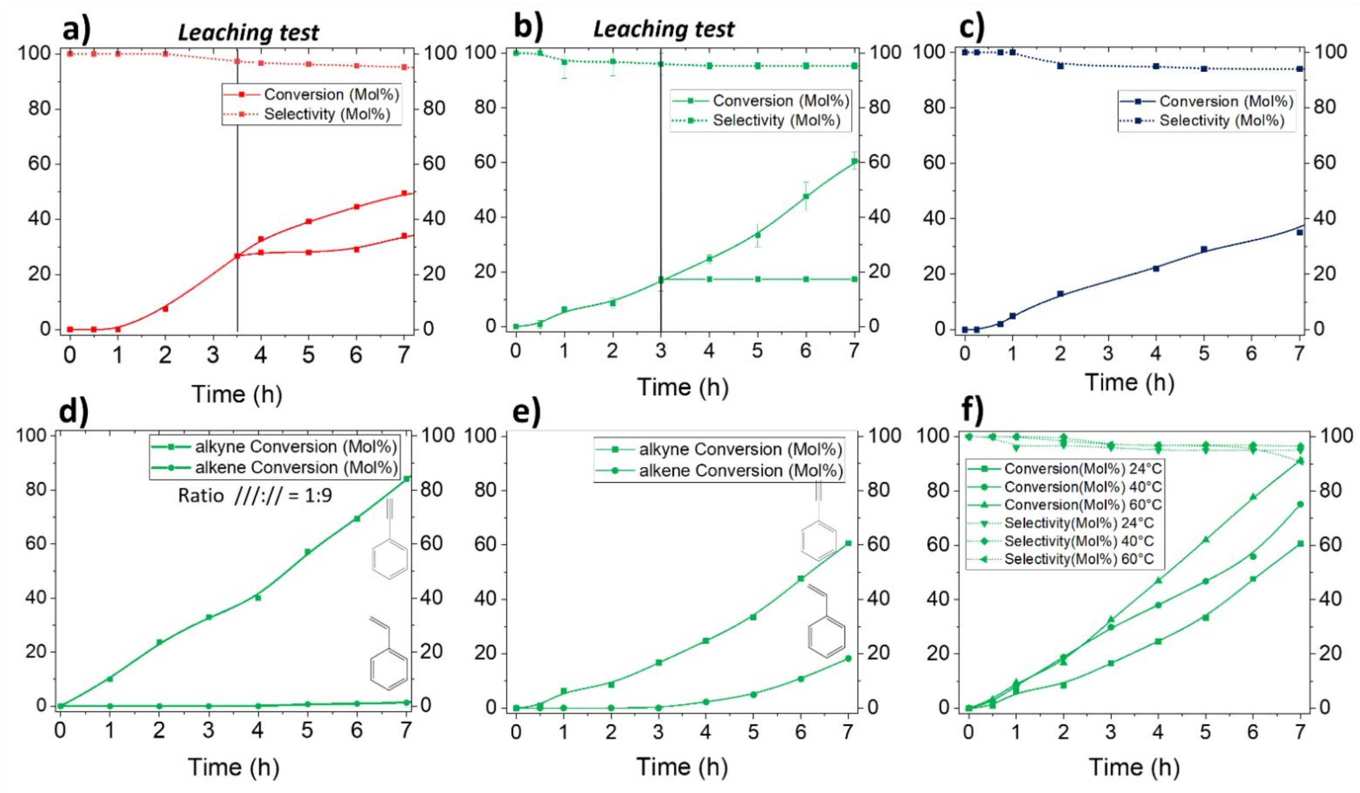

Figure S14. a), b) Kinetic curves and hot filtration of **PdIn-Q**, **PdIn-QT** catalyst and c) Kinetic curve of **PdIn-T** catalyst. d) Alkene and alkyne conversion with **PdIn-QT** catalyst during an experiment with a mix of substrate (9:1 eq respectively) e) comparison of **PdIn-QT** activity between phenylacetylene hydrogenation and styrene hydrogenation independently. f) Activity and selectivity of **PdIn-QT** catalyst at different temperature (24°C, 40°C and 60°C).

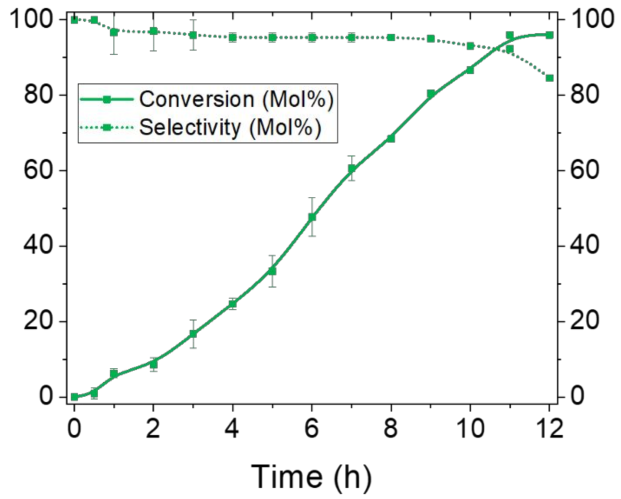

Figure S15. Conversion and Selectivity of PdIn-QT catalyst in phenylacetylene hydrogenation until 12h.

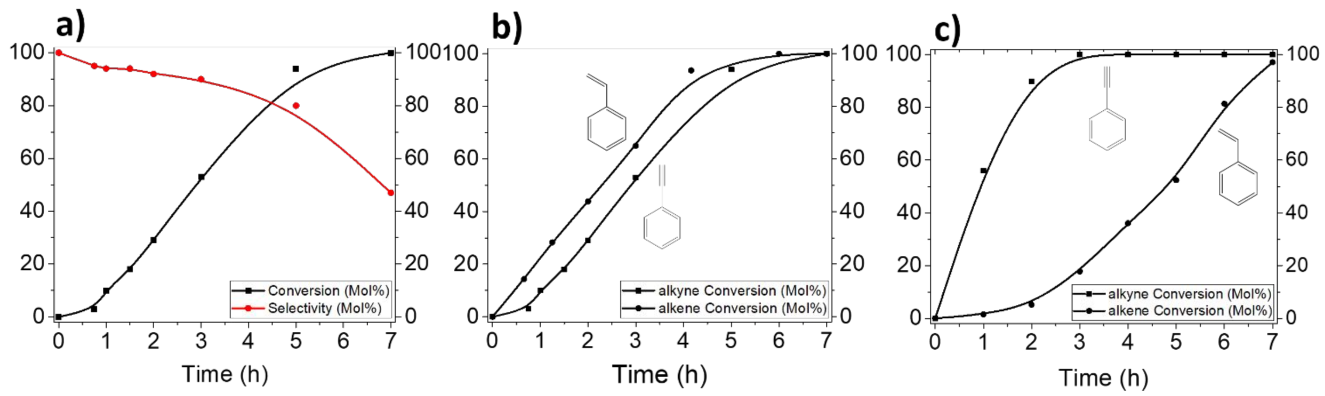

Figure S16. a) Kinetic curves of **Pd@C commercial**, b) Kinetic curve comparison of **Pd@C commercial** activity between phenylacetylene hydrogenation and styrene hydrogenation independently and c) Alkene and alkyne conversion with **Pd@C commercial** catalyst during an experiment with a mix of substrate (9:1 eq respectively).

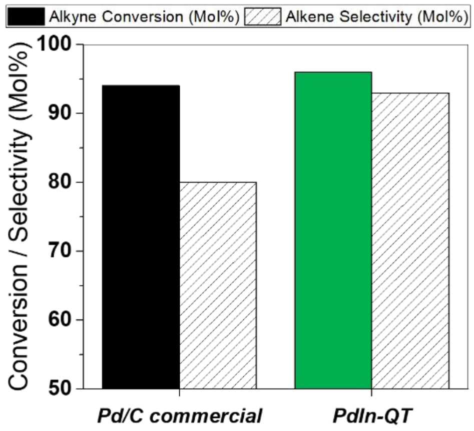

Figure S17. Activity and selectivity comparison of **Pd/C commercial and PdIn-QT at high conversion** of phenylacetylene in the semihydrogenation reaction. Reaction Conditions: 5 mmol of phenylacetylene, substrate/Pd molar ratio: 323/1, 5 mL EtOH, R.T., 1 bar H_2, 1000 rpm.

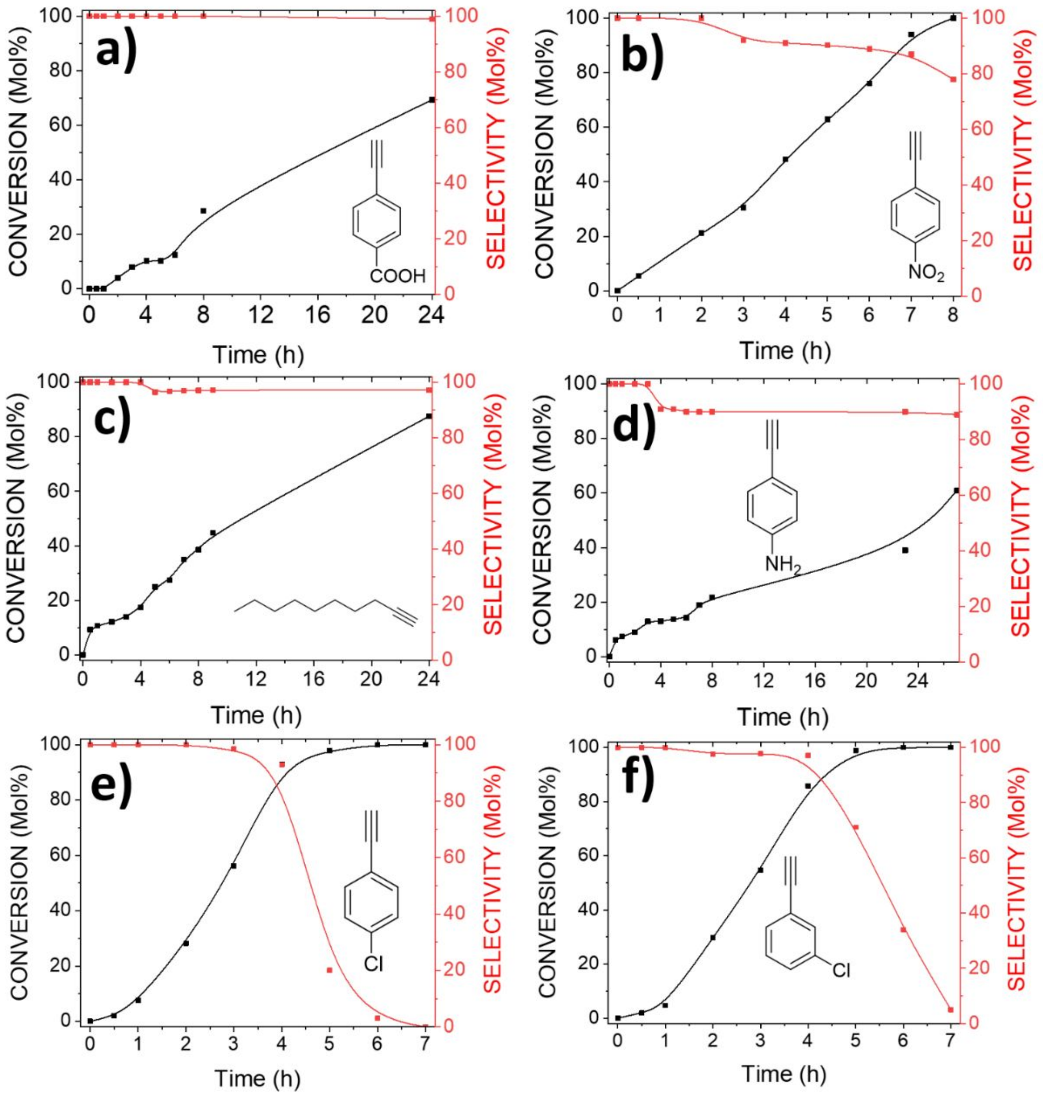

Figure S18. Conversion and Selectivity kinetic curves of **PdIn-QT** catalyst in selective hydrogenation of various alkyne compounds.

Table S4. State of the art of Pd-based monometallic and bimetallic catalysts in selective hydrogenation of phenylacetylene under analogous reaction conditions to those used in this work.

Catalyst	T (°C)	Solvent	P (bar)	C (mol%)	S (mol%)	TON	TOF (h^{-1})	Productivity ($g_{alkyne} \cdot g_{cat}^{-1} \cdot h^{-1}$)	Ref
Pd_2Ni_2/NC	*R.T.*	EtOH	1	98	95	371	279	14.6	3
PdNi@γAl_2O_3	25	EtOH	1	98	94	1485	2250	29.0	4
PdIn@Mg Al_2O_3	*R.T.*	Hexane	1	92	97	127	36	1.3	5
PdPb/C	30	CH_3CN	1	97	98	465	232	9.0	6
PdCu/NPCNs	25	EtOH	1	97	93	230	173	7.1	7
Pd-NC@NC	28	EtOH	1	68	97	73	73	0.7	8
**PdIn-QT-RUN 5**	***R.T.***	**EtOH**	**1**	**96**	**95**	**150**	**21**	**9.4**	**This work**

Note: Aimed at establishing a meaningful consideration and due to the lack of complete kinetic data, TON, TOF and productivity values were all calculated at the highest level of conversion and selectivity reported for each catalyst under consideration.

S4. Catalytic stability

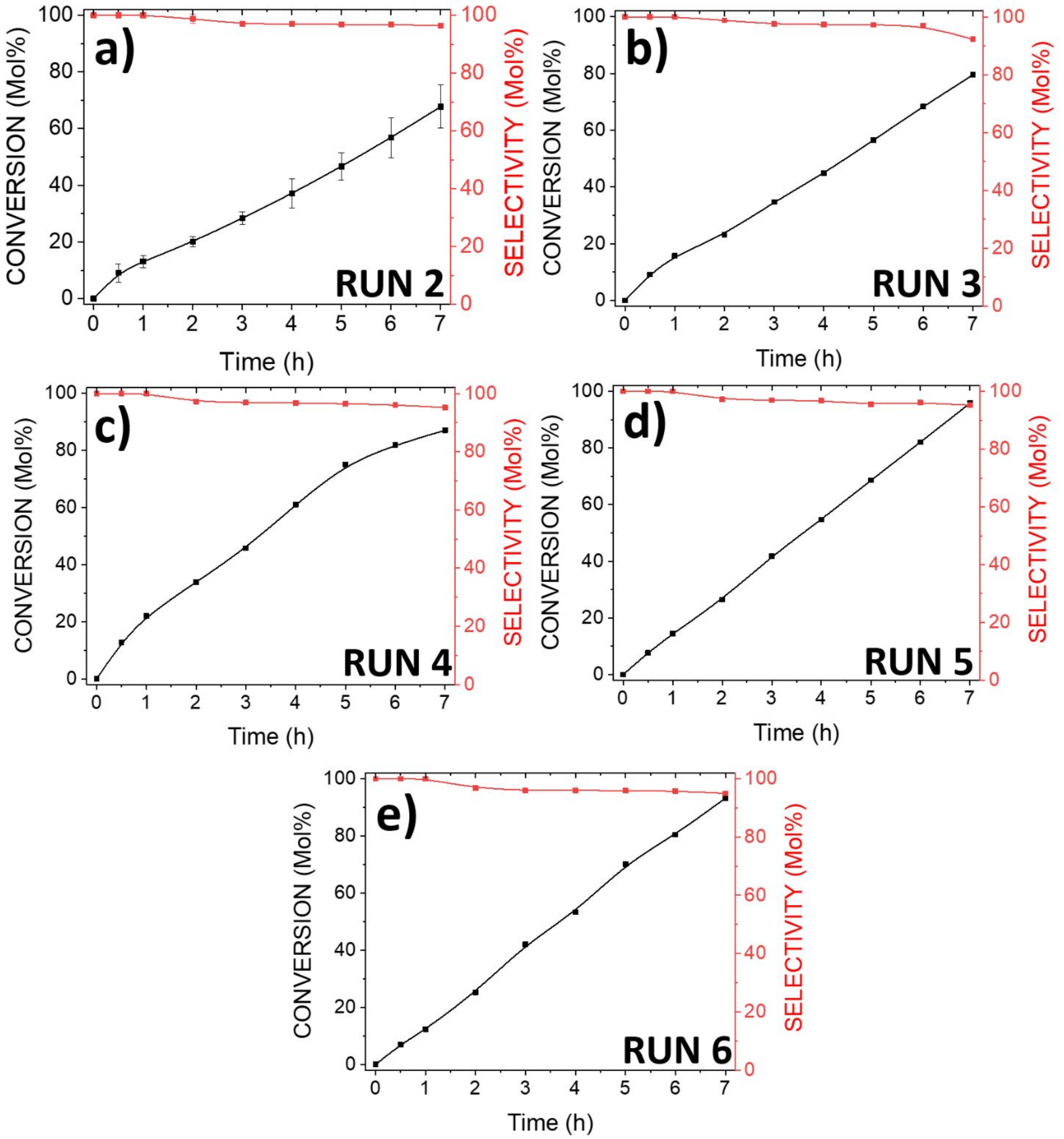

Figure S19. Conversion and Selectivity kinetic curves of **PdIn-QT** catalyst in each cycles of the stability study.

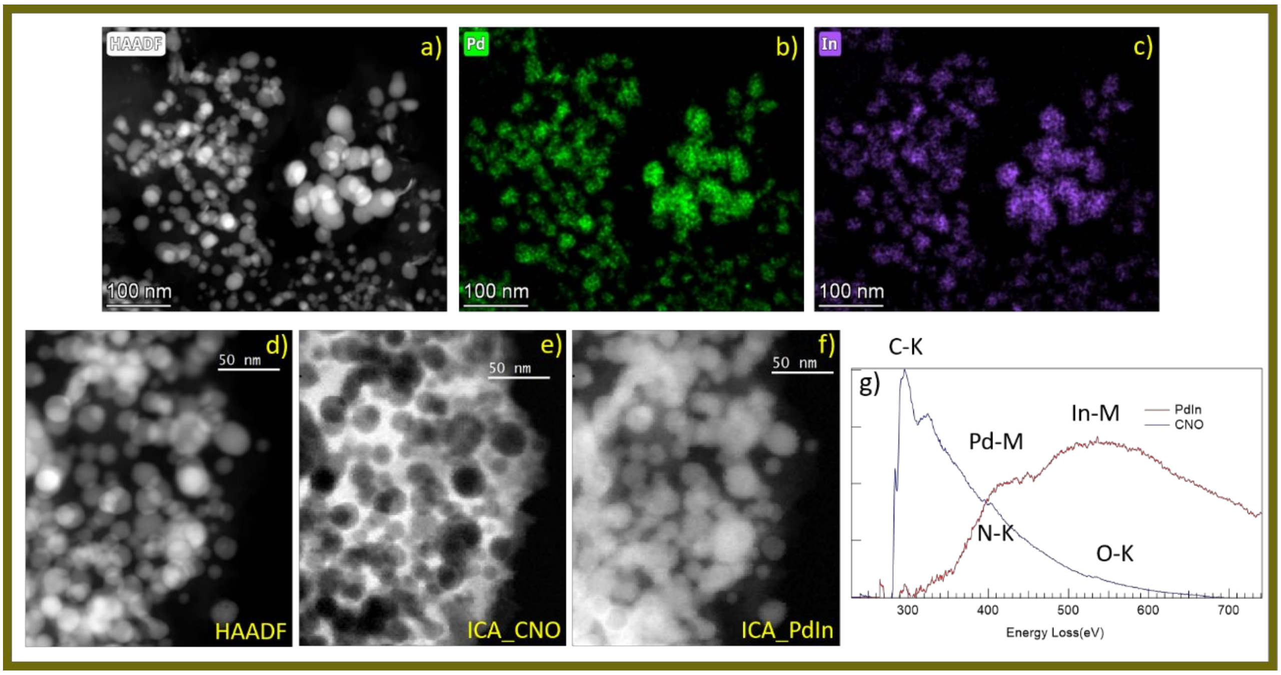

Figure S20. STEM-EDX and STEM-EELS of **PdIn-QT** after 5 catalytic cycles a) HAADF image and the corresponding chemical maps extracted from the STEM-SI-EDS Pd b) and In c). STEM-EELS study including a HAADF image d) and the images corresponding to the two components of the ICA analysis of the whole set of STEM-EELS-SI data e) and f). EELS spectra corresponding to the two independent components g), a Pd-In component and a C-N-O component.

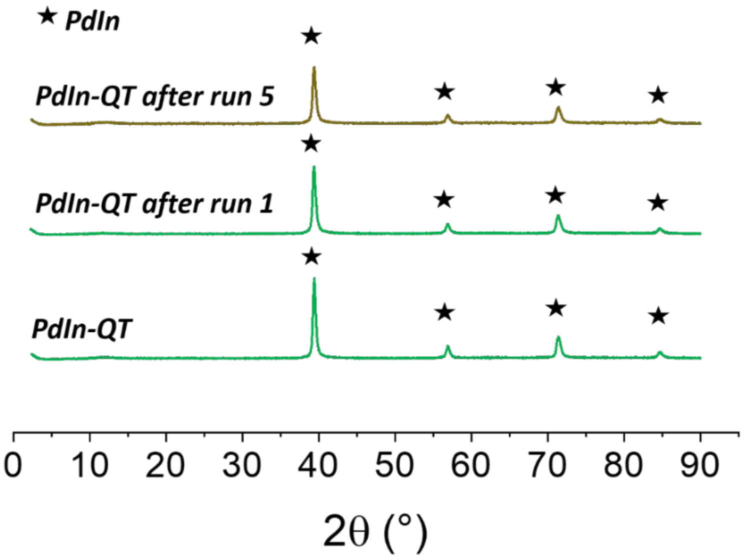

Figure S21. XRD patterns of **PdIn-QT** fresh, after 1 cycle and after 5 cycles.

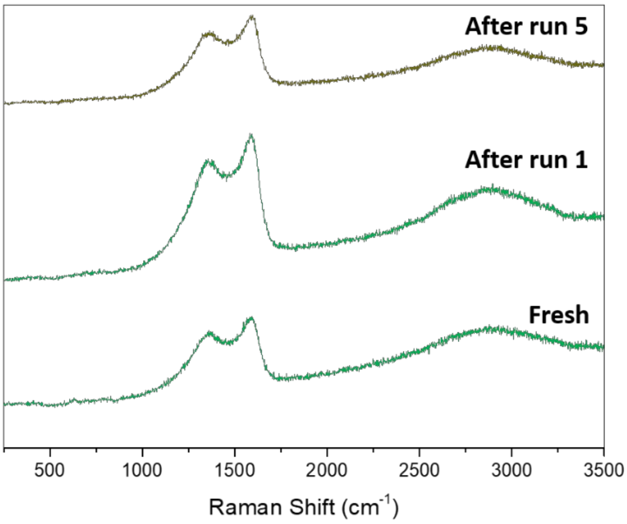

Figure S22. Raman spectra of **PdIn-QT** fresh, after 1 cycle and after 5 cycles.

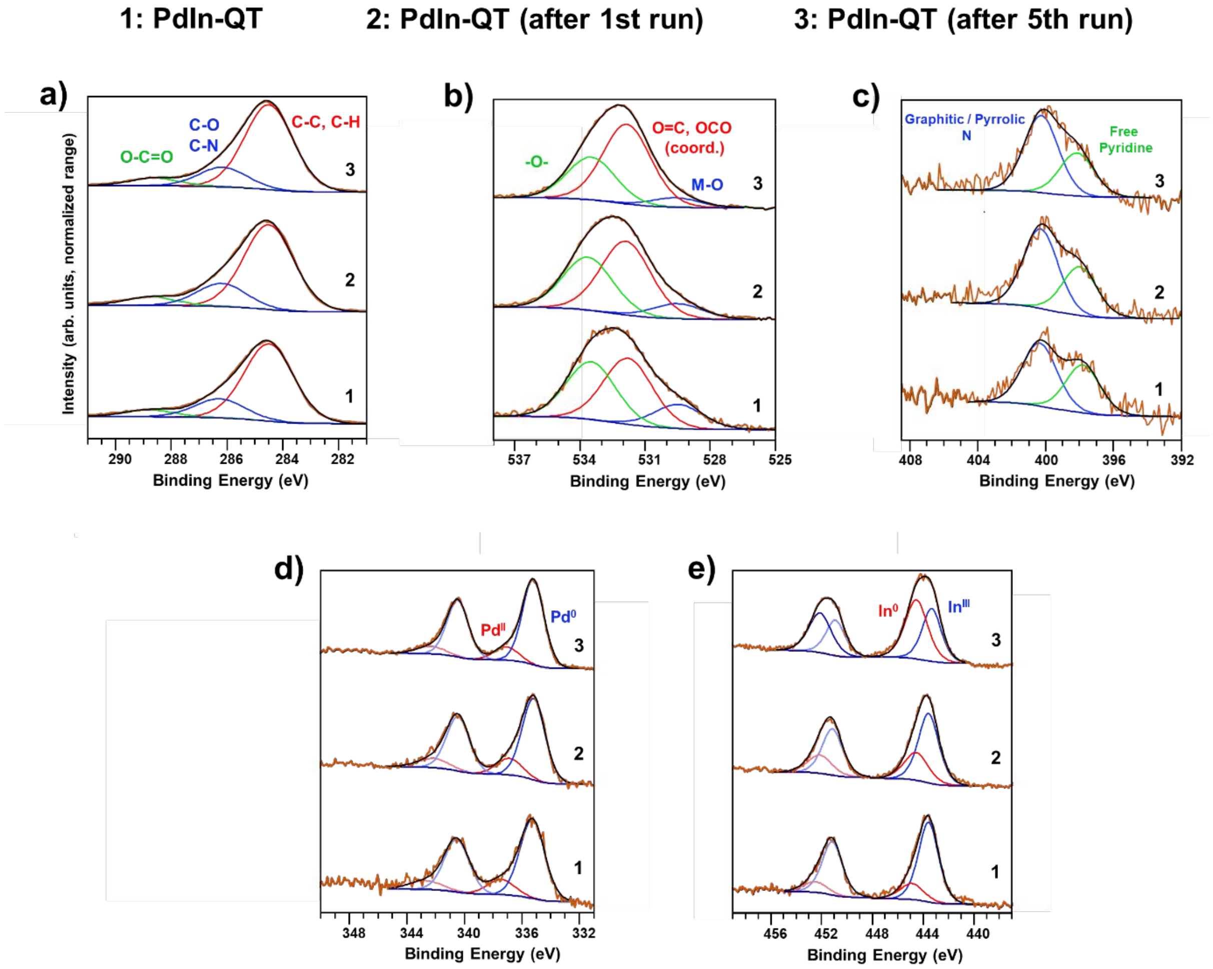

Figure S23. a) C*1s*, b) O*1s*, c) N*1s*, d) Pd*3d*, and e) In*3d* XPS regions of **PdIn-QT** fresh sample, and after 1 and 5 catalytic cycles.

ANNEX 1. XPS B.E. assignment and semi-quantitative assessment of MOF-derived PdIn samples:

Bond assignment	Material // **Elemental composition** // Position, eV (Species atomic %)* //					
	PdIn-MOF	PdIn-Q	PdIn-T	PdIn-QT	PdIn-QT (after 1st run)	PdIn-QT (after 5th run)
**C*1s***	**71.3**	**72.1**	**80.9**	**88.3**	**86.9**	**85.6**
C–C, C–H	284.5 (71)	284.5 (67)	284.5 (59)	284.5 (75)	284.5 (73)	284.5 (76)
C–N, C–O	286.0 (15)	285.9 (24)	286.3 (31)	286.3 (18)	286.2 (20)	286.2 (17)
O–C=O	288.5 (14)	288.7 (9)	288.3 (7)	288.7 (7)	288.7 (7)	288.6 (7)
Carbonate, π-electrons in aromatic ring	-	-	290.1 (3)	-	-	-
**N*1s***	**4.3**	**3.3**	**2.8**	**2.4**	**2.1**	**1.9**
Pyridinic N (uncoord.)	-	398.4 (19)	397.9 (40)	397.8 (43)	398.0 (38)	398.2 (36)
Pyridinic N (coord.)	400.1	400.0 (81)	-	-	-	-
Anilinic N (coord.)	-		-	-	-	-
Graphitic N	-	-	400.5 (60)	400.4 (57)	400.3 (62)	400.3 (64)
**O*1s***	**19.2**	**21.1**	**11.8**	**8.0**	**9.4**	**10.7**
In(μ_3-O)	528.6 (9)	528.5 (6)	-	-	-	-
M–O (M: In, Pd)	-	-	529.6 (23)	529.5 (16)	529.5 (11)	529.6 (7)
OCO (coord.), C=O	531.6 (85)	532.0 (91)	531.8 (43)	531.8 (45)	531.9 (52)	531.8 (60)
–O– (in carboxyl, H_2O or chemisorbed species)	534.6 (6)	534.5 (3)	533.9 (34)	533.5 (39)	533.6 (37)	533.5 (33)
**Pd*3d*$_{5/2}$**	**1.7**	**0.6**	**1.7**	**0.6**	**0.7**	**0.9**
Pd^0	-	335.7 (26)	335.1 (80)	335.3 (82)	335.2 (82)	335.2 (85)
Pd^{II}	-	-	337 (20)	337.4 (17)	336.9 (18)	337.1 (15)
Pd^{II} --- Cl, Pd^{IV}	338,0	338.4 (74)	-	-		
**In*3d*$_{5/2}$**	**3.5**	**2.9**	**2.8**	**0.8**	**0.9**	**0.9**
In^0	-	-	443.5 (76)	443.6 (81)	443.6 (68)	443.3 (43)
In^{III} --- O	445,0	445,4	444.7 (24)	444.9 (19)	444.5 (32)	444.5 (57)

References

(1) Spanopoulos, I.; Bratsos, I.; Tampaxis, C.; Vourloumis, D.; Klontzas, E.; Froudakis, G. E.; Charalambopoulou, G.; Steriotis, T. A.; Trikalitis, P. N. Exceptional Gravimetric and Volumetric CO_2 Uptake in a Palladated NbO-Type MOF Utilizing Cooperative Acidic and Basic, Metal–CO_2 Interactions. *Chem. Commun.* **2016**, *52* (69), 10559–10562. https://doi.org/10.1039/c6cc04790d

(2) mp-21215: InPd (Cubic, Pm-3m, 221) https://next-gen.materialsproject.org/materials/mp-21215?chemsys=Pd-In

(3) Li, J.; Kou, J.; Xiang, Y.; Chen, M.; Zhang, J.; Zhan, X.; Zhang, H.; Wang, F.; Dong, Z. ZIF-8 Derived N-Doped Porous Carbon Confined Ultrafine PdNi Bimetallic Nanoparticles for Semi-Hydrogenation of Alkynes. *Mol. Catal.* **2023**, *535*, 112865. https://doi.org/10.1016/j.mcat.2022.112865

(4) Song, X.; Shao, F.; Zhao, Z.; Li, X.; Wei, Z.; Wang, J. Single-Atom Ni-Modified Al_2O_3-Supported Pd for Mild-Temperature Semi-Hydrogenation of Alkynes. *ACS Catal.* **2022**, *12* (24), 14846–14855. https://doi.org/10.1021/acscatal.2c04883

(5) Feng, Q.; Zhao, S.; Wang, Y.; Dong, J.; Chen, W.; He, D.; Wang, D.; Yang, J.; Zhu, Y.; Zhu, H.; Gu, L.; Li, Z.; Liu, Y.; Yu, R.; Li, J.; Li, Y. Isolated Single-Atom Pd Sites in Intermetallic Nanostructures: High Catalytic Selectivity for Semihydrogenation of Alkynes. *J. Am. Chem. Soc.* **2017**, *139* (21), 7294–7301. https://doi.org/10.1021/jacs.7b01471

(6) Liu, J.; Zhu, Y.; Liu, C.; Wang, X.; Cao, C.; Song, W.; Zhu, Y.; Liu, C.; Wang, X.; ao, D.; Song, W.; Liu, J. Excellent Selectivity with High Conversion in the Semihydrogenation of Alkynes Using Palladium-Based Bimetallic Catalysts. *ChemCatChem.* **2017**, *9* (21), 4053–4057. https://doi.org/10.1002/cctc.201700800.

(7) Chen, M.; Kou, J.; Ma, H.; Xiang, Y.; Ma, P.; Sun, L.; Zhan, X.; Zhang, J.; Zhang, H.; Wang, F.; Dong, Z. Acceleration of the Semi-Hydrogenation of Alkynes over an N-Doped Porous Carbon Sphere-Confined Ultrafine PdCu Bimetallic Nanoparticle Catalyst. *Phys. Chem. Chem. Phys.* **2023**, *25* (5), 4201–4210. https://doi.org/10.1039/d2cp04845k

(8) Wang, S.; Liu, T.; Zhu, Y.; Liu, X.; Luo, Q.; Zhu, M.; Ding, T.; Yao, T. Structure-Activity Relationship of Continuous Transformed Palladium Nanoclusters and Single Atoms for Selective Phenylacetylene Hydrogenation. *J. Phys. Chem. C* **2023**, *127* (12), 5911–5919. https://doi.org/10.1021/acs.jpcc.3C00899
